# A cyclic dipeptide for salinity stress alleviation and the trophic flexibility of endophyte provide insights into saltmarsh plant–microbe interactions

**DOI:** 10.1093/ismeco/ycae041

**Published:** 2024-03-26

**Authors:** Shih-Hsun Walter Hung, Pin-Hsien Yeh, Tsai-Ching Huang, Shao-Yu Huang, I-Chen Wu, Chia-Ho Liu, Yu-Hsi Lin, Pei-Ru Chien, Fan-Chen Huang, Ying-Ning Ho, Chih-Horng Kuo, Hau-Hsuan Hwang, En-Pei Isabel Chiang, Chieh-Chen Huang

**Affiliations:** Department of Life Sciences, National Chung Hsing University, Taichung 402202, Taiwan; Institute of Plant and Microbial Biology, Academia Sinica, Taipei 115201, Taiwan; Advanced Plant and Food Crop Biotechnology Center, National Chung Hsing University, Taichung 402202, Taiwan; Department of Life Sciences, National Chung Hsing University, Taichung 402202, Taiwan; Department of Life Sciences, National Chung Hsing University, Taichung 402202, Taiwan; Department of Life Sciences, National Chung Hsing University, Taichung 402202, Taiwan; Department of Life Sciences, National Chung Hsing University, Taichung 402202, Taiwan; Department of Life Sciences, National Chung Hsing University, Taichung 402202, Taiwan; Department of Life Sciences, National Chung Hsing University, Taichung 402202, Taiwan; Department of Life Sciences, National Chung Hsing University, Taichung 402202, Taiwan; Department of Life Sciences, National Chung Hsing University, Taichung 402202, Taiwan; Institute of Marine Biology, College of Life Science, National Taiwan Ocean University, Keelung 202301, Taiwan; Centre of Excellence for the Oceans, National Taiwan Ocean University, Keelung 202301, Taiwan; Taiwan Ocean Genome Center, National Taiwan Ocean University, Keelung 202301, Taiwan; Institute of Plant and Microbial Biology, Academia Sinica, Taipei 115201, Taiwan; Biotechnology Center, National Chung Hsing University, Taichung 402202, Taiwan; Department of Life Sciences, National Chung Hsing University, Taichung 402202, Taiwan; Advanced Plant and Food Crop Biotechnology Center, National Chung Hsing University, Taichung 402202, Taiwan; Innovation and Development Center of Sustainable Agriculture, National Chung Hsing University, Taichung 402202, Taiwan; Advanced Plant and Food Crop Biotechnology Center, National Chung Hsing University, Taichung 402202, Taiwan; Innovation and Development Center of Sustainable Agriculture, National Chung Hsing University, Taichung 402202, Taiwan; Department of Food Science and Biotechnology, National Chung Hsing University, Taichung 402202, Taiwan; Department of Life Sciences, National Chung Hsing University, Taichung 402202, Taiwan; Advanced Plant and Food Crop Biotechnology Center, National Chung Hsing University, Taichung 402202, Taiwan; Innovation and Development Center of Sustainable Agriculture, National Chung Hsing University, Taichung 402202, Taiwan

**Keywords:** endophyte, cyclic dipeptide, molecular plant–microbe interactions (MPMI), symbiosis

## Abstract

In response to climate change, the nature of endophytes and their applications in sustainable agriculture have attracted the attention of academics and agro-industries. This work focused on the endophytic halophiles of the endangered Taiwanese salt marsh plant, *Bolboschoenus planiculmis*, and evaluated the functions of these isolates through *in planta* salinity stress alleviation assay using *Arabidopsis*. The endophytic strain *Priestia megaterium* BP01R2, which can promote plant growth and salinity tolerance, was further characterized through multi-omics approaches. The transcriptomics results suggested that BP01R2 could function by tuning hormone signal transduction, energy-producing metabolism, multiple stress responses, etc. In addition, the cyclodipeptide cyclo(L-Ala-Gly), which was identified by metabolomics analysis, was confirmed to contribute to the alleviation of salinity stress in stressed plants via exogenous supplementation. In this study, we used multi-omics approaches to investigate the genomics, metabolomics, and tropisms of endophytes, as well as the transcriptomics of plants in response to the endophyte. The results revealed the potential molecular mechanisms underlying the occurrence of biostimulant-based plant-endophyte symbioses with possible application in sustainable agriculture.

## Graphical Abstract

**Graphical Abstract:**
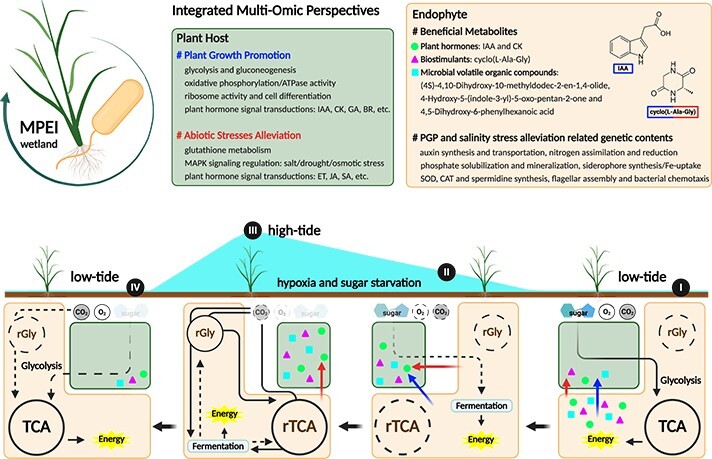

## Introduction

Climate change and projected world population growth are challenging for our food supply systems [1, 2]. To ensure adequate food security and sustainability, it is crucial to develop new technologies for growing crops efficiently in extreme environments [3]. In addition to traditional breeding and precision genetic technologies, plant growth-promoting rhizobacteria (PGPRs) and endophytes have also been implemented to enhance crop nutrient and water absorption, hormone signal transduction, stress adaptation, etc. [4, 5]. Among them, endophytes that live inside plants (in the endosphere) are attracting increased attention due to their *in planta* colonization properties and specific effects on host plants for growth promotion and tolerance to both abiotic and biotic stresses [6–9]. Various endophytes isolated from diverse origins have been demonstrated to exhibit positive effects by producing bioactive metabolites known as biostimulants [10–15].

Endophytes isolated from saline habitats, such as salt marshes or alkaline lakes, may be promising candidates for endophyte-assisted agriculture in saline or overfertilized fields. For the isolation of such endophytic halophiles, plants growing in conditions with continuously high salinity and periodic hypoxia are suitable sources [16–18]. Salt marsh plants often face multiple stresses, but many wetland-related studies have been limited to microbe-assisted phytoremediation [19–22]. Therefore, understanding the strategies used by these unique plant-associated microbes living in stressful environments through symbiotic mechanisms may provide novel knowledge and tools for future applications.

In previous studies, endophytic strains were isolated from various natural habitats and used to construct synthetic plant-endophyte symbiosis systems for plant cultivation under biotic or abiotic stress conditions [5, 23–26]. On the other hand, several specific metabolites, such as pyrroloquinoline quinone and cyclodipeptides (CDPs), have been identified as potential biostimulants [23, 27–30], and some CDPs have also been reported as agricultural agents that can protect crops against biotic and abiotic stresses [31–35]. Although some of them had been patented for applications more than three decades ago [36], the molecular bases of the role of CDPs in moderating plant stresses and in plant–microbe interactions remain to be elucidated.

In this study, halotolerant and PGP endophytes were isolated and characterized from endangered salt marsh plants in Taiwan. The complete genome and metabolome of *Priestia megaterium* BP01R2 (previously also known as BP-R2), along with its *in planta* regulations on host transcriptome, were further investigated to reveal the molecular mechanism involved in such symbiotic relationships. In addition, the CDP cyclo(L-Ala-Gly) was identified as a novel CDP biostimulant for plant salinity stress alleviation. The results of this study facilitate our understanding of the enigmatic cross-kingdom host–microbe interactions and unveil promising endophytic biostimulants for sustainable agriculture.

## Materials and methods

### Plant materials and sampling

**Figure 1:**
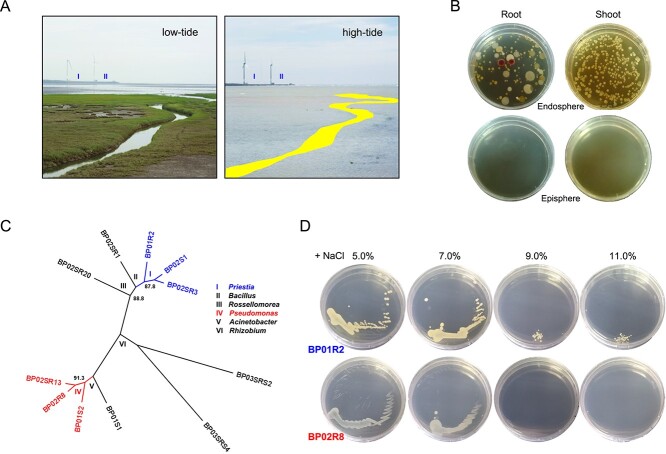
**Typical habitat and identification of bacterial endophytes in *Bolboschoenus planiculmis*; A** typical niche, tidal marsh in Taichung, Taiwan at low- (left) and high-tide (right); I and II show two corresponding locations in two different photographs; the pseudo-colour marks indicate a channel visible in the low-tide; **B** endophytic microbiome; bacterial endophytes isolated from different plant tissues (endosphere); the last rinsing solution used for surface sterilization was set as control checks (episphere); **C** unrooted 16S rRNA gene-based maximum likelihood phylogeny of eleven bacterial candidates; all internal nodes received >85% bootstrap support based on 1000 re-samplings; the roman numerals indicate that the groups belong to different genera; the groups containing isolates BP01R2 and BP02R8, which were further focused on in this work, are shown in blue and red, respectively; **D**  *in vitro* salinity stress tolerance test of endophytes.

The plant material (*Bolboschoenus planiculmis*) for endophyte isolation was collected from a tidal marsh in Taichung, Taiwan (Fig. 1A). The seeds of *Arabidopsis thaliana* ecotype Columbia (Col-0) were retrieved from Chieh-Chen Huang Lab’s stock.

### Endophyte isolation, characterization, and 16S ribosomal RNA gene-based phylogeny

Endophytic bacteria were isolated and maintained as described in our previous works [23, 26]. In brief, the endosphere fluids of surface-sterilized plant tissues were subjected to endophyte isolation and incubation using Luria-Bertani (LB) agar or broth at 37°C for 24–48 h. Isolates were first picked based on their different morphologies, and a preliminary taxonomic check was conducted via 16S ribosomal RNA (rRNA) gene-based phylogeny. Bacterial indole-3-acetic acid (IAA) production was examined as described by Hwang *et al*. [26]. The 16S rRNA genes were amplified using the primer set E8F/U1510R as previously described [5, 26]. Multiple sequence alignments of 16S rRNA genes were performed using MUSCLE v3.8.31 [37]. Maximum likelihood phylogenies were inferred using PhyML v3.3 [38] and visualized using FigTree v1.4.4. PHYLIP v3.697 [39] was used for bootstrap analysis.

### Plant-endophyte symbiosis and biostimulant assays

Unless otherwise stated, *Arabidopsis* plants were grown according to previously described methods [26, 40]. In short, seeds were germinated on Murashige and Skoog (MS) plates supplemented with NaCl (ranging from 0 to 170 mM) under a 10/14 h day/night cycle at 25°C. Endophytes were incubated and inoculated to the plants as described in our previous work [26]. Briefly, strains BP01R2 and BP02R8 were adjusted on OD_600_ = 0.8, ≈ 3.5 × 10^7^ and 9.4 × 10^8^ CFU/ml, respectively, and the strains were serially diluted into OD_600_ = 0.4 and 0.04 as the relatively high concentration inocula (HCI) and low concentration inocula (LCI) for the symbiosis assays. The biostimulant cyclo(L-Ala-Gly) (SS-2476, Combi-Blocks; San Diego, CA, USA) was purchased from UNI-ONWARD Corp (New Taipei City, Taiwan) and was prepared for the plant experiments.

### Plant total RNA extraction and transcriptomic analysis

*Arabidopsis* seedlings grown on MS plates supplemented with 100 mM NaCl and inoculated with or without strain BP01R2 (OD_600_ = 0.8 ≈ 3.5 × 10^7^ CFU/ml; prepared as above mentioned) were harvested 15 days after inoculation (DAI); those grown on MS plates without NaCl supplementation were used as controls. Samples were homogenized with a mortar and pestle using liquid nitrogen; the total RNA was extracted from the mixtures of five seedlings in each treatment using TRIzol reagent. All procedures were performed following Welgene Biotech's in-house pipeline and our previous works on plant transcriptomics [41, 42]; all kits were used according to the manufacturer's instructions, and all bioinformatics tools were used with the default settings.

Briefly, library construction was carried out with Agilent's SureSelect Strand-Specific RNA Library Preparation Kit with poly-A and size selection using oligo(dT) beads and AMPure XP beads (Beckman Coulter; Chaska, MN, USA), respectively. The 75 bp single-end sequencing was performed on the Illumina Solexa platform. The Illumina program bcl2fastq v2.20 was used for basecalling, and low-quality reads were trimmed off based on Q20 accuracy using Trimmomatic v0.36 [43]. The resultant sequence was mapped to the TAIR10 genome [44] using HISAT2 aligner [45, 46], and the differential expression analysis was performed using StringTie v2.1.4 [47, 48] and DEseq2 v1.28.1 [49] with genome bias detection/correction and Welgene Biotech's in-house pipeline. The transcript per million (TPM) method was used for normalization, and genes with low expression levels (TPM <0.3) in either or both of the treated and control samples were excluded from the analysis [50]. Genes with 2.0-fold TPM differences and a probability of at least 0.95 were defined as differentially expressed genes (DEGs). Gene set enrichment analysis [51] was carried out for the enrichment analysis, and the DEGs were subjected to Gene Ontology (GO) [52] and Kyoto Encyclopedia of Genes and Genomes (KEGG) [53] database for metabolic pathway prediction.

### Bacterial metabolomics

The strain BP01R2 was cultured on LB agar plates with or without NaCl, as mentioned above. Five agar plates were collected for each sample, and metabolites were extracted with ethyl acetate two times. The extracts were re-dissolved in methanol and adjusted to 10 mg/ml. All samples were analysed using the linear ion trap mass spectrometer system (LTQ XL, Thermo Fisher Scientific; San Jose, California, USA) with direct injection at positive ion mode. The mass range was from m/z 100–1500. MS raw data files were converted to mzXML format using MSConvert [54]. Metabolite peak detection was performed using MZmine version 2.53 [55], and the pre-processed data were conducted by R program (version 4.0.3) [56]. The pre-processed MS feature table and unknown metabolites were searched and annotated against referenced metabolites of the AntiMarin database [57] by exact molecular mass to identify the molecular formula and for annotation of discriminating features.

### Bacterial genome sequencing, assembly, and annotation

The procedures for genome sequencing and analysis were based on those described in our previous work on bacterial genomes [29, 58]. All kits used were the same as previously described and were used according to the manufacturer’s protocols, and all bioinformatics tools were used with the default settings unless stated otherwise. Briefly, the total DNA of strain BP01R2 was extracted and then sequenced via Illumina NovaSeq 6000 2 × 150 bp paired-end and Oxford Nanopore Technologies (ONT) MinION platforms. Further filtering was conducted to remove ONT reads shorter than 12 000 bp. A hybrid *de novo* assembly was produced using Unicycler v0.4.9-beta [59]. For validation, the Illumina and ONT raw reads were mapped to the assembly using BWA v0.7.17 [60] and Minimap2 v2.15 [61], respectively. The results were programmatically checked with SAMtools v1.9 [62] and manually inspected using IGV v2.11.1 [63]. For assembly completeness evaluation, benchmarking universal single-copy orthologous (BUSCO v5.1.2) analysis, referenced to the Bacillales dataset, was executed on gVolante [64, 65]. The finalized assembly was submitted to the National Center for Biotechnology Information (NCBI) and annotated using the Prokaryotic Genome Annotation Pipeline (PGAP) [66]. KofamKOALA [67] was used to examine putative metabolic pathways.

### Bacterial comparative genomics

All BioProject and BioSample records of *P. megaterium* accessible on NCBI as of 28 February 2022 were retrieved for comparative analysis with BP01R2 (Table S1). FastANI v1.1 [68] was used for whole-genome comparison to calculate the proportion of genomic segments mapped and the average nucleotide identity (ANI). Homologous gene clusters were identified using OrthoMCL [69] for gene content analysis. Genes associated with plant growth-promoting (PGP) traits and rhizosphere competence were identified according to previous works on *P. megaterium* [70] and bacterial endophytes [71]; genes associated with salinity stress [72–74] and autotrophs [75–78] were also identified. Multiple sequence alignments of homologous genes, maximum likelihood phylogenies, and bootstrap analysis were conducted based on the methods described for 16S rRNA gene phylogeny.

### Bacterial tropism analysis

Anaerobic cultivation was performed according to the instructions of the Leibniz Institute DSMZ [79]. The Hungate anaerobic culture tube and Coy anaerobic chamber (Coy Laboratory Products, USA) were used in this experiment. BP01R2 was precultured in M9 minimal medium (248510; Becton Dickinson, USA) for 24 h, and then washed three times using the same method before use as an inoculant. The inoculant density was set at OD_600_ = 0.01; the cultivation volume was 5 ml, and the culture was carried out by shaking at 120 rpm and 37°C. M9 minimal medium supplemented with 2 mM MgSO_4_ (131-00405; FUJIFILM Wako, Japan), 0.1 mM CaCl_2_ (21 075; Sigma-Aldrich, USA), 10 mM NaNO_3_ (195-02545; FUJIFILM Wako, Japan), and 0.01% L-tryptophan (A10230; Alfa Aesar, USA) was prepared as a general incubation medium. Extra glucose was tested as an organic carbon resource at concentrations (w/v) of 0.4%, 0.2%, 0.04%, and 0%; the tube headspace gas was composed of either sterile air or 90% H_2_ plus 10% CO_2_ (N_2_). Growth curves were measured at OD_595_ using the PARADIG detection platform (Beckman, USA) or a Sunrise absorbance microplate reader (Tecan, Austria).

### Statistics

Shapiro–Wilk and Levene’s tests were used to check the normal distribution of variables and homoscedasticity. A parametric one-way analysis of variance analysis with Tukey's post hoc honestly significant difference (HSD) test or non-parametric Kruskal–Wallis test with Dunn's post hoc test was then applied to evaluate statistical significance. Data analysis was performed using the Real Statistics Resource Pack v9.0 [80]. For all experiments, at least five independent biological replicates were tested, and the data are shown as mean ± SEM unless otherwise stated. For all data points, the different letters indicate statistical significance between the samples at ^*^P ≤ 0.05 level; otherwise, not significant.

**Figure 2:**
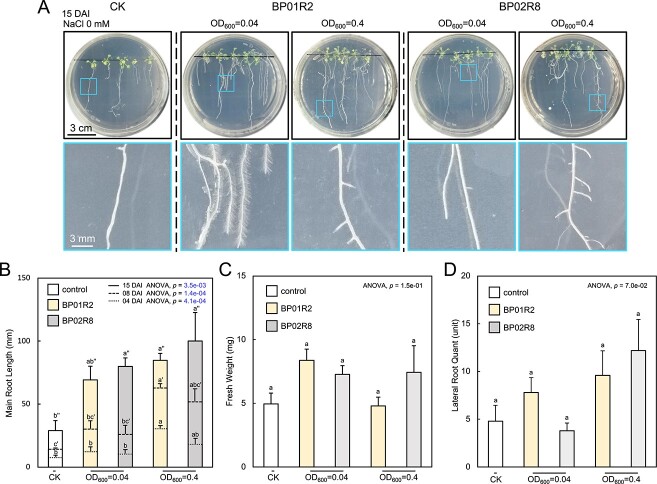
**Endophytes promote host plant growth; A** plant growth promotion differs with inoculation concentration and type of endophyte; the upper five panels illustrate the plant growth patterns under treatment with different inocula, and the lower five panels provide a close-up view of the roots; CK: control check; DAI: days after inoculation; **B** the main root length, **C** fresh weight, and **D** lateral root number of different inoculum-treated plants; the data are presented as mean ± SEM; *N* = 5; the different letters indicate statistical significance between samples (^*^P < 0.05); otherwise, not significant.

## Results

### Isolation of halotolerant bacterial endophytes

In total, 128 and 519 colony-forming units (CFUs) were isolated from the endospheres of roots and shoots, respectively; however, the microbiome composition in the shoots was less diverse (Fig. 1B). Among the picked isolates, 11 endophytes consisted of six genera: *Priestia*, *Pseudomonas*, *Rhizobium*, *Bacillus*, *Acinetobacter*, and *Rossellomorea* (ordered from high to low enrichment) (Fig. 1C). A *Priestia* sp. strain (designated as strain BP01R2) and a *Pseudomonas* sp. strain (designated as strain BP02R8) were chosen for the following experiments due to their enriched distribution and presence of beneficial endophytes affiliated with these genera [70, 81, 82]. According to the results of the halotolerance test, both strains tolerate 7.0% NaCl supplementation, and BP01R2 can even survive at an extremely high concentration of 11% NaCl, which was therefore identified as a halophile (Fig. 1D).

### Plant-endophyte symbiosis benefits *in planta* salinity stress alleviation

The symbiosis assay was first tested without salinity stress. Both BP01R2 and BP02R8 presented plant-growth-promotion characteristics when prepared as either LCI or HCI (Fig. 2A). Both strains induced the formation of plant lateral roots under HCI (Fig. 2A and D), and BP01R2 caused more root hairs and fresh weight under LCI (Fig. 2C). Besides, the main root length was also determined at 4, 8, and 15 DAI. The results showed that BP02R8 significantly increased the main root length at 15 DAI either under LCI or HCI, while BP01R2 contributed to the main root elongation at all time points under HCI with statistical significance (Fig. 2B). The main root length was thus considered a leading indicator to evaluate the endophytic contribution and was then focused on in the following experiments. These results echoed previous observations of the ability of these strains to promote plant growth [26] and highlighted their potential benefits for rhizosphere competence.

**Figure 3:**
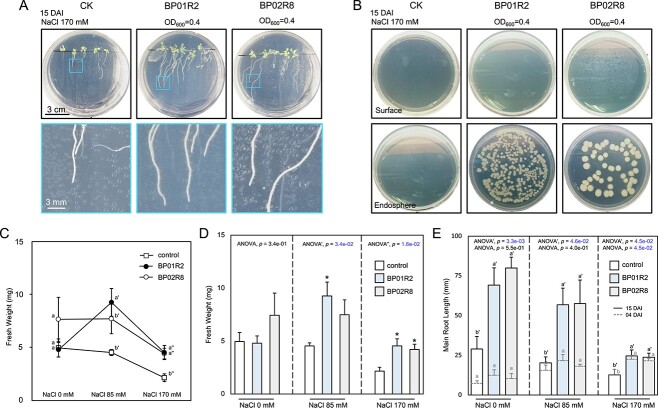
**Endophytes alleviate salinity stress in host plants; A** plant growth under endophytic inoculation and salinity stress; the upper three panels illustrate the plant growth patterns with different inocula under salinity stress, and the lower three panels show close-up images of the roots; CK: control check; DAI: days after inoculation; **B** endophyte reisolation; **C, D** the fresh weight (15 DAI), and **E** main root length (04 and 15 DAI) of plants treated with different inocula under salinity stress; the data are presented as mean ± SEM; *N* = 5; the different letters indicate statistical significance between samples (^*^P < 0.05); otherwise, not significant.

The seedlings of *A. thaliana* were subsequently grown in MS media supplemented with 85 or 170 mM NaCl for 15 days to mimic salinity stress. BP01R2 and BP02R8 promoted plant growth on shoots, roots, and fresh weight, similar to the findings under stress-free conditions (Fig. 3). Phenotypes of chlorotic and adaxial-curled leaves, fewer lateral roots and shorter main root length observed in control plants (CK) under 170 mM NaCl stress were mitigated through endophyte inoculation (Fig. 3A). For instance, the main root length decreasing percentage was recovered from 56.2% (CK) to 15.2% (BP01R2) and 17.9% (BP02R8), respectively (Fig. 3E). Both endophytes significantly increased the main root length and fresh weight at 15 DAI under 170 mM NaCl stress, and BP01R2 also contributed the same under 85 mM NaCl stress with statistical significance (Fig. 3C–E).

### Cyclo(L-ala-Gly) improves plant growth and alleviates *in planta* salinity stress

**Figure 4:**
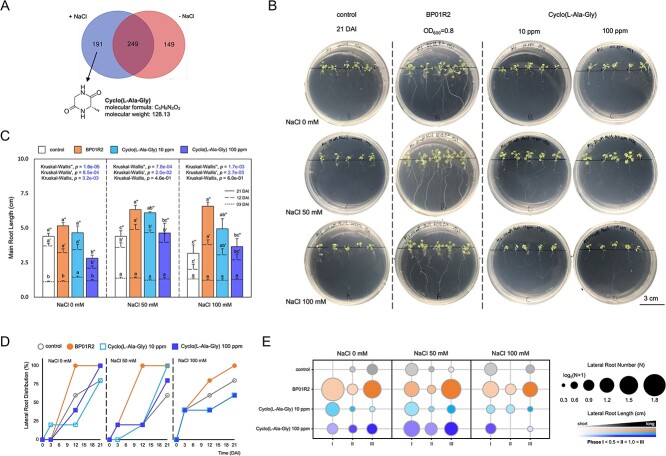
**Metabolomic analysis of BP01R2 and the biostimulant candidate for mitigating *in planta* salinity stress; A** Cyclo(L-Ala-Gly) was identified under NaCl-specific bacterial growth conditions; **B** plant growth with BP01R2 and cyclo(L-Ala-Gly) inoculations under salinity stress; the lower and upper four panels are the plants grown with 50 or 0 mM NaCl, respectively; CK: control check; DAI: days after inoculation; **C** main root length; the dotted, dashed, and solid lines indicate 03, 12, and 21 DAI, respectively; **D** lateral root emergence ratio between 03, 12, and 21 DAI; the open and closed circles and squares indicate the control, BP01R2, cyclo(L-Ala-Gly) 10 ppm and cyclo(L-Ala-Gly) 100 ppm, respectively; **E** the quant and length distribution of lateral roots at different phases; a greater circle size indicates a greater lateral root number; lateral root developmental phases I, II, and III indicate the lateral root lengths <0.5 cm, between 0.5 and 1.0 cm, and > 1.0 cm, respectively, and correspond to the colour keys; the data were collected from five independent seedlings with three technical repeats and are presented as mean ± SEM; the different letters indicate statistical significance between samples (^*^P < 0.05); otherwise, not significant.

To investigate which metabolites contribute to the salinity stress alleviation in hosts, a linear ion trap mass spectrometer system was used to analyse the metabolomics of BP01R2 under NaCl-present and NaCl-absent incubation conditions. In total, 191 compounds were identified specifically under the NaCl-present condition, and 249 compounds were found to be shared by NaCl-present and NaCl-absent conditions (Fig. 4A and Tables S4 and S5). Among the profiles, 18 cyclic dipeptides, also known as 2,5-diketopiperazines and CDPs, were identified (Table S6). These CDPs could be classified into proline-based (including hydroxyproline) and non-proline-based groups, and nine were observed only under the NaCl-present condition. CDPs have been reported to act as biostimulants against biotic and abiotic stresses in plants [31–35], and many proline- and hydroxyproline-based CDPs have been shown to induce resistance in plants against abiotic stresses [36]. However, the understanding of those non-proline-based CDPs and their roles in molecular plant–microbe interactions (MPMI) is limited. Therefore, the only non-proline-based CDP, cyclo(L-Ala-Gly), produced by BP01R2 under NaCl-present condition, was targeted for the follow-up biostimulants assays.

After 3 weeks of exogenous application of cyclo(L-Ala-Gly) to plants (i.e. 21 DAI to BP01R2), the 10 ppm (C10) or 100 ppm (C100) supplementation contributed to main root elongation and lateral root development but not to shoot growth. Still, apparent contributions to the alleviation of salinity stress (i.e. vigorous growth of seedlings and the alleviation of leaf chlorosis and adaxial curling) were observed under 50 mM NaCl conditions (Fig. 4B). The decrease in plant main root length was attenuated by BP01R2, C10, and C100 application under 50 and 100 mM NaCl. BP01R2 and C10 benefited the main root elongation under 0 mM NaCl. All treatments had similar effects under 100 mM NaCl conditions (Fig. 4C). Furthermore, the lateral root formation rate (number of plants with lateral roots/number of plants without lateral roots), number, and length were calculated to estimate the salinity stress alleviation by the treatments. BP01R2 and C100 showed greater lateral root formation rates than CK under 0 mM NaCl. All treatments led to greater lateral root formation than CK under 50 mM NaCl. Only BP01R2 had greater lateral root formation than CK and maintained a 100% lateral root formation rate under 100 mM NaCl (Fig. 4D). More lateral roots were observed in all the treatments than in the CK under 0 and 50 mM NaCl conditions, and only BP01R2 maintained this advantage under 100 mM NaCl condition (Fig. 4E, shown as circular sizes).

For lateral root length, all samples were classified by length to represent three different developmental phases (phases PI, PII, and PIII indicate the lateral root lengths <0.5 cm, between 0.5 and 1.0 cm, and > 1.0 cm, respectively) (Fig. 4E, referred as colour keys). The results showed that the PII and the PIII of C100 were longer than CK under 0 mm NaCl. Both C10 and C100 increased the lateral root length at different phases under 50 mM NaCl. No obvious contributions were found from C10 or C100 under 100 nM NaCl (Fig. 4E). Overall, C10 and C100 contributed to plant growth and stress alleviation by promoting lateral root formation and elongation as observed under 0 and 50 mM NaCl conditions. These results suggested that cyclo(L-Ala-Gly), along with other metabolites, may contribute to BP01R2 in alleviating *in planta* salinity stress and improving plant growth. This indicated its nature as a novel biostimulant, which has already been patented (Taiwan Intellectual Property Office assigned number I684411).

### Plant transcriptomics in response to endophytic symbiosis

**Figure 5:**
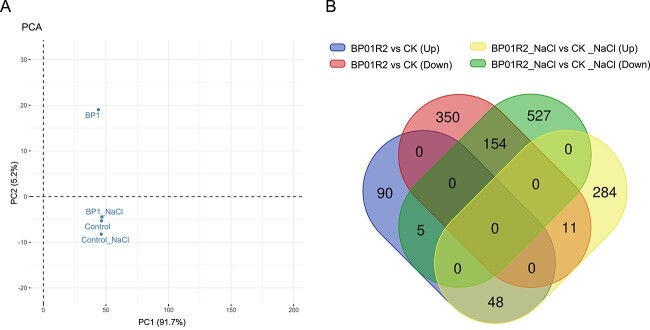
**Transcriptomic analysis; A** principal component analysis for transcriptomic datasets; **B** Venn diagram of DEGs comparison between samples with or without salinity stress.

Given the characteristics of BP01R2 in alleviating salinity stress in plants presented in previous [26] and this work (Figs 3 and 4), we subsequently collected the plant RNA from the above experimental batches to study the potential molecular regulations of BP01R2 to host plants underlying the salinity stress. A total of 30 956 567 (~2.3 Gb), 34 330 286 (~2.6 Gb), 31 539 938 (~2.4 Gb), and 32 554 093 (~2.4 Gb) trimmed reads were generated for CK, BP01R2, CK_NaCl, and BP01R2_NaCl, respectively. The sequence mapping rate against the *Arabidopsis* reference genome ranged from 98.41% to 99.23%. Strikingly, the expression pattern of the CK was intermediate to the samples that suffered from salinity stress, and the BP01R2_NaCl showed a higher similarity to the CK instead of the CK_NaCl (Fig. 5A), which may result from the alleviation contributed by the symbiosed endophyte as seen in the phenotypic data (Fig. 4B–E).

When the symbiosis occurred without salinity stress, a total of 658 DEGs among the 21 028 genes were identified (Fig. S1A), and the GO analysis revealed the corresponding enrich-regulated terms of biological processes (plant epidermal and root cell differentiation and root morphogenesis), cellular component (chromosome, cytoskeleton, transferase, and cytoplasmic vesicle), and molecular function (oxygen binding, ATPase activity, helicase activity, and ligase activity) (Figs S2 and S3). Consistent with the GO enrichment results, several PGP-related pathways (e.g. glycolysis and gluconeogenesis, oxidative phosphorylation, plant hormone signal transduction, etc.) were also shown to be enrich-regulated within KEGG analysis results (Figs S4–S7). These enriched gene regulations well explained the phenotypes observed in the symbiosis assays (i.e. the growth improvement traits such as main root elongation, lateral root induction, fresh weight accumulation, etc.) (Figs 2 and 3A and C–E).

When the symbiosis occurred under salinity stress, a total of 1029 DEGs were identified among the 20 937 genes (Fig. S1B). The cell death, hypersensitive response, jasmonic acid biosynthesis and response, ethylene response, MAPK signalling, and glutathione metabolism, among others, were up-regulated (Figs S8–S13), with results usually opposite compared with those of the CK_NaCl and CK comparisons (Figs S14–S17). Moreover, the plant hormone signal transduction and ribosome-related pathways were consistently up-regulated, similar to what was observed in symbiosis without the salinity stress. Furthermore, the simultaneously up- (co-up-DEGs) and down-regulated DEGs (co-down-DEGs) between samples with and without salinity stress were also examined. A total of 202 co-DEGs were universally up- or down-regulated, regardless of salinity stress or not (Fig. 5B). Among them, the 48 co-up-DEGs were found to be involved in plant-type cell wall organization or biogenesis, xyloglucan:xyloglucosyl transferase activity, peroxidase activity, oxidoreductase activity, antioxidant activity, etc. (Table S2); the 154 co-down-DEGs were enriched in response to hypoxia, jasmonic acid, salicylic acid, abscisic acid, oxidoreductase, oxidative stress, and related metabolism (Table S3). In summary, the transcriptome data are consistent with the observed phenotypes and explain at the molecular level how BP01R2 symbiosis alleviates *in planta* salinity stress and promotes the host plant growth.

### Genome assembly and comparative genomics of BP01R2 and its relatives

**Figure 6:**
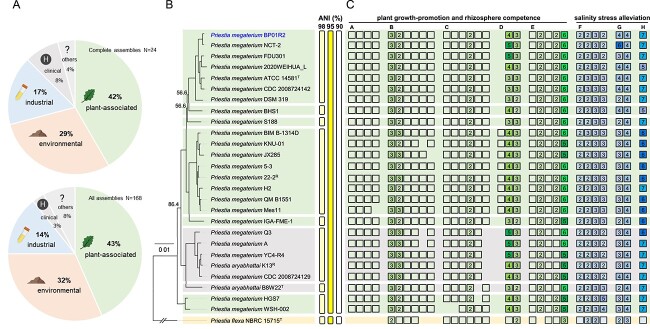
**Evolutionary relationships among *Priestia megaterium* BP01R2 and its relatives; A** source origin distribution of all available *Priestia megaterium* genomes; **B** phylogeny of strain BP01R2 and its relatives; the maximum likelihood phylogeny is based on the concatenated alignment of 2155 single-copy genes shared by all strains analysed (147 671 aligned amino acid sites); the bootstrap values of those three internal nodes with <99% were labelled; *Priestia flexa* NBRC 15715^T^ is included as the outgroup; the superscripts “T” and “R” following the strain names indicate type strains and strains assigned as NCBI representative genomes, respectively; strains with complete genomes are presented in bold, and the strain BP01R2 reported in this work is highlighted in blue; information to the right of the phylogenic tree shows the grouping of genomes according to different cut-off values of genome-wide ANI; **C** gene content analysis; the empty box and box with numbers inside indicate single-copy and multiple-copy of the targeted genes, respectively; the different genes are clustered in groups: A, nitrogen assimilation and reduction (*nasA-E*); B, phosphate solubilization and mineralization (*gcd, phoP, phoR, phoAB, phoD* and *ppx*); C, L-tryptophane and indole synthesis (*trpA-F*); D, IAA synthesis and transporter (*gatA* and an AEC family transporter encoding gene); E, acetoin and butanediol synthesis (*alsD, alsS, ilvH,* and *ilvG*); F, spermidine synthesis (*speAB* and *speDE*); G, superoxide dismutase and catalase (*sodACF and cat*); H, ferredoxin (*fer*) related; the gene locus accession details are provided in Table S7; the icons were created with BioRender.com.

**Figure 7:**
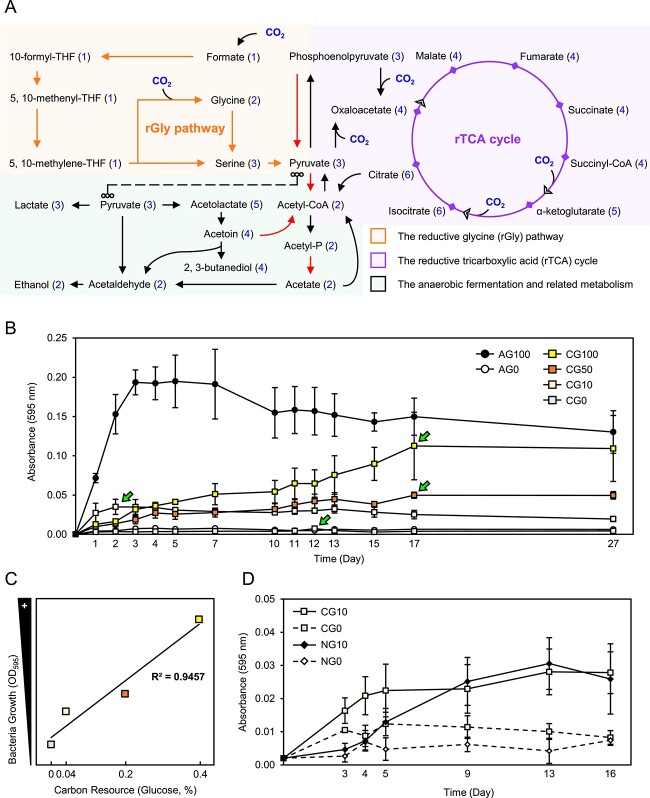
**Tropism analyses on BP01R2; A** simplified autotrophic (orange and purple panels)- and anaerobic (green panel)-related metabolism identified within the BP01R2 genome; all of the gene names and locus accession details are listed in Table S7; the carbon dioxide (CO_2_) influx and the number of carbon atoms in each molecule are highlighted in blue; the reactions involved in energy generation are marked as red arrows; **B**, **D** bacterial growth curves under different carbon sources (G0, G10, G50, and G100 indicate 0%, 0.04%, 0.2%, and 0.4% glucose, respectively) and tube headspace gas compositions (A, C, and N indicate sterilized air, 90% H_2_ plus 10% CO_2_, and 90% H_2_ plus 10% N_2_, respectively); **C** coefficient relationship between carbon resources and bacterial growth under anaerobic conditions; X-axis, glucose content; Y-axis, the maxima (indicated by the fluorescent green arrows) of the bacterial growth curves in **B**; all the data points were collected from three independent biological replicates and shown as mean ± SEM.

The genome of strain BP01R2 was sequenced to investigate the key genes for its functional characterization and evolution. In total, 2 × 6486 500 (∼2.0 Gb) of Illumina and 26 722 (~0.4 Gb; N_50_: 13469 bp) of trimmed ONT raw reads were obtained for hybrid *de novo* genome assembly. The Illumina and ONT reads provided 349.5X and 66.7X coverage, respectively. The assembly result indicated that BP01R2 has one circular chromosome (5228 948 bp) with 38.1% GC content and six plasmids with sizes ranging from 5296 to 134 664 bp. For the completeness evaluation, 449 complete BUSCOs (99.8%), one fragmented BUSCO (0.2%), and no missing BUSCOs (0.0%) were found within this assembly, which is consistent with the expectation that circular chromosomal contig represents the complete genome assembly. The annotation contains 15 complete sets of rRNA genes and two additional 5S rRNA genes, 157 tRNA genes, eight non-coding RNAs, 5551 protein-coding genes, and 91 pseudogenes (Table 1). To investigate the genes associated with the molecular plant–endophyte interactions (MPEI), genes involved in (a) plant growth promotion and rhizosphere competence, (b) salinity stress alleviation, and (c) carbon and oxygen limitation adaptation were identified and further discussed for their potential contribution to the symbiotic relationships (Figs 6, 7, and S19).

**Table 1:** Genomic features of *P. megaterium* strain BP01R2.

**Features**	**Chromosome**	**pBP01R2a**	**pBP01R2b**	**pBP01R2c**	**pBP01R2d**	**pBP01R2e**	**pBP01R2f**
**Accession**	CP092387.1	CP092388.1	CP092389.1	CP092390.1	CP092391.1	CP092392.1	CP092393.1
**Size (bp)**	5228 948	134 664	133 343	50 994	39 414	12 668	5296
**GC content (%)**	38	34	34.5	35.5	36.5	34	36
**Protein-coding genes**	5231	108	119	44	31	10	8
**Pseudogenes**	82	3	4	1	0	1	0
**Ribosome RNA genes**	40	0	0	4	3	0	0
**Transfer RNA genes**	121	0	1	17	18	0	0
**Non-coding RNA genes**	8	0	0	0	0	0	0

For the species assignment, we calculated the genome-wide ANI of BP01R2 and its relatives (Table S1). Additionally, 2155 single-copy genes shared by all strains were identified and used to produce a concatenated alignment containing 147 671 aligned amino acid sites for maximum likelihood phylogeny construction. The results showed that BP01R2 is closely related to *P. megaterium* NCT-2, and all of the ingroup genomes share >95% ANI threshold recommended for bacterial species delineation [68] (Fig. 6B). Based on this, strain BP01R2 was identified as *P. megaterium*. The BioProject and BioSample records of all *P. megaterium* available from NCBI were also examined and checked for their source origin distribution. As results show, over half of *P. megaterium* were isolated from plant-associated or environmental sources (Fig. 6A). Many of them were reported as beneficial endophytes, PGP bacteria, or originally inhabited in alkaline and hypersaline environments (e.g. salt marshes, alkaline lakes, potash salt dumps, etc.), suggesting their immense potential in dealing with such abiotic stresses [83–85].

For focused gene content investigations, the genes related to MPMI and salinity stress alleviation (Fig. 6C) were further examined. BP01R2 encodes 180 proteins commonly associated with nutrient acquisition, phytohormone production, rhizosphere competence, and the ability against abiotic stress in host plants (Fig. S19 and Table S7). Genes encoding ABC transporters (Fig. S20) and two-component systems (Fig. S21) are enriched in the BP01R2 genome and likely related to the processing of environmental information. For rhizosphere competence, five of the *nar*, *nas,* and *nir* genes related to nitrogen assimilation and reduction [86, 87], as well as a *gcd* gene, a *ppx* gene, and nine *pho* genes regulating phosphate solubilization, transport, and assimilation [88, 89] were found. Additionally, a total of 30 protein-encoding genes associated with flagellar biosynthesis and assembly (i.e. *flh*, *fli*, *flg*, and *mot* genes), eight *che* genes related to bacterial chemotaxis, and three *als*, three *ilv*, and six *bdh* genes related to bacterial PGP volatiles (e.g. acetoin and butanediol) [90–92] are present within this genome (Table S7).

For plant growth improvement, 11 genes were found to be involved in the indole-3-acetamide pathway for IAA biosynthesis from tryptophan [91, 93], which was consistent with the metabolomic results for IAA production. Moreover, four auxin efflux carrier (AEC) family transporter protein-encoding genes [94, 95] and 10 genes encoding siderophore synthesis/Fe-uptake proteins possibly making BP01R2 capable of producing siderophores that could assist host plants in chelating ferric ions under iron starvation [87, 96] were identified (Fig. 6C and Table S7). Concerning the usual hypoxia and hyper-alkaline/saline conditions in a typical niche of BP01R2 and its plant host, abundant genes involved in the reductive tricarboxylic acid (rTCA) pathway [76, 77], the reductive glycine (rGly) pathway [78], and anaerobic respiration metabolism were characterized (Fig. S19 and Table S7). Additionally, four *sod* genes encoding superoxide dismutase family proteins, five *cat* genes encoding catalase proteins, and nine *spe* genes involved in spermidine synthesis related to *in planta* salinity stress alleviation [97] were also found in this genome (Fig. 6C and Table S7).

### Identification of mixotrophic characteristics

The BP01R2 genome contains the general nitrite reductase genes *nirBD*, which are required for nitrate ammonification [98], the lactate dehydrogenase gene *ldh*, which primarily mediates NAD^+^ regeneration during lactate fermentation [99]; and the required genes involved in the other conversion processes, such as the pyruvate-acetoin-2,3-butanediol and the pyruvate-acetyl-CoA-acetate reaction [75, 100] (Figs 7A and S19 and Table S7). Moreover, the anaerobic regulation related *resA-E* operon encoded [101, 102] and the *fnr* gene were also found [103] (Table S7). Notably, two nonspontaneous bacterial carbon fixation machineries, the rGly pathway [78] and the rTCA cycle [76, 77], are present in this genome (Figs 7A and S19, and Table S7). Furthermore, except for three industrial isolates, genes encoding the key enzymes of these systems (e.g. *gcvHT* for the rGly pathway and *korAB* for the rTCA cycle) were also found within all the examined *Bacillus megaterium* genomes (including the type strain), suggesting the potentials for trophic flexibility in this species to assimilate inorganic carbon sources from the environment. To estimate the anaerobic and autotrophic availability of BP01R2, it was incubated under oxygen and carbon resource starvation conditions by mimicking the original habitat of its photoautotrophic host. The results showed that BP01R2 is able to grow under anaerobic conditions (Fig. 7B), and a high correlation (R^2^ = 0.95) was found between the extra glucose supplementation and the maximum bacterial growing capability (Fig. 7C), indicating the ability of BP01R2 to acquire organic carbon resources for growth under anaerobic conditions.

Furthermore, we were interested in whether BP01R2 uses CO_2_ as a sole carbon resource to grow under anaerobic respiration conditions as a chemolithoautotroph (H_2_ as an electron donor, NaNO_3_ as an electron acceptor, and CO_2_ as a carbon source). The tested glucose concentrations were 0.04% and 0%, and N_2_ was used to replace the CO_2_ in anaerobic gas. The results confirmed that BP01R2 can use CO_2_ for growth either with or without extra 0.04% glucose (Fig. 7D). Interestingly, the bacteria grew significantly better with CO_2_ than N_2_ on the third day (CG0 vs NG0, *P* = 0.05; CG10 vs NG10, *P* = 0.03), and this advantage was maintained when the bacteria were growing without glucose until the 16th day. A similar pattern was found under the 0.04% glucose conditions before the ninth day, and a greater growth rate was observed in the CO_2_ treatments; however, no significant difference was found in the maximum bacterial growing capacity compared with that under N_2_ conditions after then. Accordingly, BP01R2 was identified as a facultative aerobic mixotroph that can use organic (glucose) or inorganic (CO_2_) carbon resources to grow.

## Discussion

### BP01R2 and cyclo(L-Ala-Gly) as versatile endophytic biostimulants

Auxin is well known as a crucial instructor of root/shoot morphogenesis and development by converging multiple phytohormones and signalling pathways in plants [104–106]. In this work, several IAA-producing genes and AEC family transporter genes were identified within the genome of BP01R2 (Table S7), and its IAA production was also confirmed either at NaCl-absent (Fig. S18 and Table S5) or NaCl-present conditions (Table S4) [26]. In addition, several PGP bacterial volatiles and (lateral) root-inducing biostimulants (Tables S4 and S5) and more than 40 genes related to bacterial flagella and chemotaxis (Table S7) were identified in the BP01R2 genome. Some endophytes have bacterial flagella and produce biostimulants, which cause different repulsive or attractive compounds and quorum sensing. The contact between endophytes and plant exudates occurs accordingly, and chemotaxis driven by flagella also plays an important role in colonization [96, 107–109].

**Figure 8:**
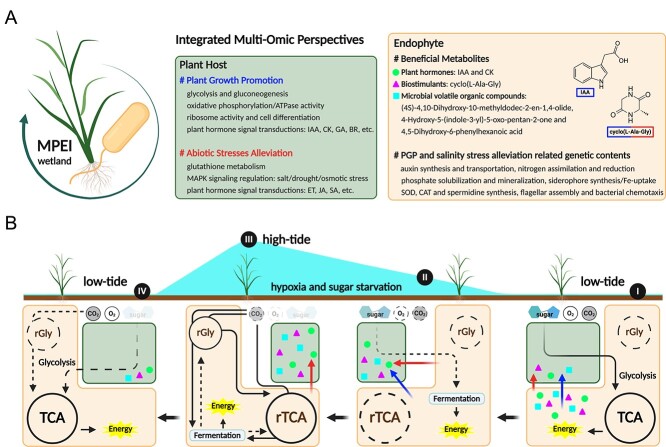
**Schematic illustration of MPEI among wetland biomes; A** integrated multi-omics perspective on the symbiotic relationships of wetland plants and endophytes; the green cells indicate enriched phenotypic and transcriptomic regulations within the plant contributed by the endophyte; the orange cells were generated based on metabolomic and genomic data analyses in this work; **B** the dynamic carbon resource acquisition switch of the endophyte may constitute a niche for host-symbiosis under hypoxia and sugar starvation; for simplicity, the black arrow represents any carbon flux from organic (sugar) or inorganic (CO_2_) carbon sources; both dashed lines and transparent marks indicate a decreasing level of suitability or availability; phases I, II, III, and IV indicate four timepoints among the tidal dynamics of the wetland biome, and the diagrams below indicate the timepoint-specific MPEI accordingly; the blue and red marks highlight the plant growth promotion and abiotic stress alleviation effects on the plant host, respectively; the brightly coloured symbols indicate the corresponding beneficial metabolites mentioned in the orange cell of **A**; icons were created with BioRender.com.

The resulting root structure changes to maintain morphological plasticity, preventing the salt accumulation of plants from saline environments [110, 111]. In addition to phytohormones, multiple pathways, such as glutathione metabolism and ribosome biosynthesis, respond in an opposite pattern to those without stress [112–114]. Similar phenomena were also found for BP01R2 inoculated plants (Figs 3 and S10–S17), suggesting its ability to alleviate *in planta* salinity stress. While investigating bacterial metabolomics under salinity stress, several cyclic dipeptide signals were identified, and cyclo(L-Ala-Gly) was later confirmed to function like BP01R2 in plants (Fig. 4). In contrast to our previous work describing the PGP traits and application [26], in this study, we deciphered the genome and metabolome of BP01R2, and examined the plant transcriptome to reveal the molecular mechanisms of its roles in MPMI and *in planta* stress alleviation. Taken together, the findings suggest that BP01R2 improves plant growth and alleviates salinity stress with beneficial biostimulants and transcriptomic regulations in host plants (Fig. 8A).

Although the potential of CDPs as stress-mitigating biostimulants, especially cyclo(L-Ala-Gly), was revealed in this work, further investigations are required to decipher their molecular roles in plants. The functions and the molecular mechanisms of these compounds in plants have yet to be elucidated; one of the reasons is that developing an efficient and specific method for detecting their *in planta* amount and distribution is technically difficult. In summary, this work facilitates our understanding of CDPs and highlights the importance of these potent small compounds, which deserve additional attention.

### Taxonomy of *Priestia* species

Based on information available for the strains with NCBI BioProject/BioSample information, >70% of *P. megaterium* strains are associated with plants or are environmental microbes (Fig. 6A), and all of these strains have abundant PGPR and stress abatement-related genetic components (Fig. 6C), suggesting the potential of this species for agricultural applications. Based on genome-wide ANI analysis and the molecular phylogeny of conserved single-copy genes, some of the characterized strains may be misclassified. Notably, our results showed that the type strain of *P. aryabhattai* shares >95% ANI with *P. megaterium* (Fig. 6B). To clarify the species assignments, we backtracked the supporting information described for the delineation of these species [115]. However, two conserved signature indels reported by Gupta *et al*. [115] were absent in neither the type strain of *P. megaterium* nor *P. aryabhattai* (Fig. S22), suggesting that these markers are not reliable. Based on the International Code of Nomenclature of Prokaryotes (Principle 8, “Each order or taxon of a lower rank with a given circumscription, position, and rank can bear only one correct name, i.e. the earliest that is in accordance with the Rules of this Code”) [116], we recommend rejecting the name *Priestia aryabhattai* (Basonym: *Bacillus aryabhattai* [117]) and temporarily replacing it with *Priestia megaterium* (Basonym: *B. megaterium* [118]). This taxonomic reclassification coincides with the synonymy suggestion by Narsing Rao *et al*. [119]. Moreover, further confirmation of the correctness and suitability of the name *Priestia* gen. nov. is required to avoid incorrect use.

### Insights into symbiosis among wetland MPEI

As mentioned, BP01R2 originally inhabited salt marsh plants that grow under continuous high salinity and periodic hypoxia (Fig. 1A). When plants experience abiotic stress, e.g. salinity stress, drought stress, and osmotic stress, the transmissible sugars in plants tend to accumulate in the vacuole for water pressure manipulation or be recycled into stored forms of sugar (e.g. starch, cellulose, lignin, etc.), and microorganisms living in the plant endosphere may thus suffer *in planta* carbon resource starvation [120–122]. There is consensus that efficient carbon resource transmission (i.e. sugars, amino acids, other organic matters, etc.) from plant phloem and root exudates to symbionts is important [123, 124]. Therefore, addressing these routine multi-stress conditions is crucial for maintaining such symbiosis among the endosphere. The genomic analysis (Figs 7A and S19 and Table S7) and tropism analysis (Fig. 7B–D) of BP01R2 revealed its availability on anaerobic and autotrophic metabolisms and mixotrophic traits, allowing it to grow under conditions *in vitro* mimicking wetland plants suffering the multi-stress of sugar starvation and hypoxia.

Overall, we infer that the anaerobic and autotrophic characteristics of the endophyte may reduce the carbon source demand from the host under hypoxia stress, such as during the high-tide period in wetlands (from Phase I to Phase III, Fig. 8B), i.e. the pathway by which strains gain energy might be able to be switched from consuming sugar and the TCA cycle to anaerobic carbon fixation, such as the rTCA cycle, and fermentation (Phase III, Fig. 8B). These may constitute a strategy and a niche for symbionts to increase their competitiveness for host symbiosis under multiple stresses or within extreme biomes. Based on multi-omics investigations and dynamic switching of carbon resources and energy acquisition of *P. megaterium* BP01R2, a novel perspective on MPEI in the wetland biome is proposed (Fig. 8). However, the coordination of carbon resource acquisition (organic and inorganic) and trophic patterns (heterotrophic and autotrophic) among such MPEI has not been fully elucidated. Research on multi-dimensional regulations in plant–microbe interactions [125–127] and tracing fluxes of carbon resources among bacteria and plant hosts [128] are necessary for future work.

## Conclusions

Beyond uncovering the versatile potential of *P. megaterium* BP01R2 and cyclo(L-Ala-Gly) for agricultural purposes, the multi-omics and bacterial tropism results presented in this work provide new insights into the unclear mechanism of trophic homeostasis among MPEI. Multitudes of microorganisms colonize diverse compartments of healthy plants, and their importance in the modulation of commensal or mutual relationships has been widely discussed [124, 129, 130]. Nevertheless, how bacterial tropism shapes the autotrophic and energy metabolisms of endophytes, either through transcriptomic or metabolic dynamics, to maintain their competitiveness among host–microbe symbiosis systems remains obscure. The symbiosis of mixotrophs and photoautotrophs reported herein provides a preliminary but novel perspective and untapped opportunities to understand the enigmatic plant–endophyte symbiotic relationships.

## Supplementary Material

Table_S1237_ycae041

Table_S456_ycae041

Fig_S1toS22_ycae041

## Data Availability

The complete genome sequence of *Priestia megaterium* BP01R2 has been deposited in GenBank under the accession numbers CP092387 (chromosome) and CP092388–93 (plasmids). The genome and RNA sequencing project and the associated raw reads were deposited in the NCBI under BioProject PRJNA806882 and PRJNA818431, respectively.
