# Supplementary material for: A cyclic dipeptide for salinity stress alleviation and the trophic flexibility of endophyte provide insights into saltmarsh plant–microbe interactions
Source: ISME Commun. 2024 Mar 26;4(1):ycae041. doi: 10.1093/ismeco/ycae041 (PMC11070113; doi:10.1093/ismeco/ycae041)
Supplement: Table_S1237_ycae041 [file table_s1237_ycae041.pdf]

ISME Communications Supporting Information

Article title:

A cyclic dipeptide for salinity stress alleviation and the trophic flexibility of an endophyte reveal niches in salt marsh plant-microbe interactions

Authors:

Shih-Hsun Walter Hung[1,2,3], Pin-Hsien Yeh[1], Tsai-Ching Huang[1], Shao-Yu Huang[1], I-Chen Wu[1], Chia-Ho Liu[1], Yu-Hsi Lin[1], Pei-Ru Chien[1], Fan-Chen Huang[1], Ying-Ning Ho[4,5], Chih-Horng Kuo[2,6], Hau-Hsuan Hwang[1,3,7], En-Pei Isabel Chiang[3,7,8] and Chieh-Chen Huang[1,3,7*]

Affiliations:

1 Department of Life Sciences, National Chung Hsing University, Taichung 402202, Taiwan

2 Institute of Plant and Microbial Biology, Academia Sinica, Taipei 115201, Taiwan

3 Advanced Plant and Food Crop Biotechnology Center, National Chung Hsing University, Taichung 402202, Taiwan

4 Institute of Marine Biology, College of Life Science, National Taiwan Ocean University, Keelung 202301, Taiwan

5 Center of Excellence for the Oceans, National Taiwan Ocean University, Keelung 202301, Taiwan

6 Biotechnology Center, National Chung Hsing University, Taichung 402202, Taiwan

7 Innovation and Development Center of Sustainable Agriculture, National Chung Hsing University, Taichung 402202, Taiwan

8 Department of Food Science and Biotechnology, National Chung Hsing University, Taichung 402202, Taiwan

*Corresponding authors: Chieh-Chen Huang, cchuang@dragon.nchu.edu.tw

Table S1. List of the genome sequences used in this study. Species name abbreviations: *Pm*, *Priestia megaterium*; *Pa*, *Priestia aryabhattai*; *Pf*, *Priestia flexa.* Superscripts following the strain names: T, type strain; R, NCBI representative genome. Category abbreviations: P, plant-associated; I, industrial; C, clinical; E, environmental; O, others. NA indicate the data are missing or inaccessible.

Species	Strain	Accession	Assembly	Size (Mb)	GC (%)	Geographic origin	Isolation source	Category
Pm	BP01R2	GCA_022537925	Complete	5.61	37.50	Taichung, Taiwan	Endosphere of *Bolboschoenus planiculmis*	P
Pm	ATCC 14581^{T}	GCA_017086525	Complete	5.75	37.85	NA	NA	O
Pm	S2	GCA_012275205	Complete	6.47	38.60	Russia	NA	I
Pm	CDC 2008724142	GCA_017086565	Complete	6.00	37.78	Rhode Island, USA	*Homo sapiens*	C
Pm	BHS1	GCA_015582655	Complete	5.37	37.80	Giresun city, Turkey	Alkaline water	E
Pm	2020WEIHUA_L	GCA_022023815	Complete	5.92	37.56	China	Soil	P
Pm	S188	GCA_011058275	Complete	5.41	37.91	Cheonan-si, South Korea	Soil	E
Pm	FDU301	GCA_013146705	Complete	6.87	36.89	Shanghai, China	Paper surface	E
Pm	Mes11	GCA_013458535	Complete	6.17	37.55	Limagne, France	Agricultural soil growing maize	P
Pm	BIM B-1314D	GCA_013389435	Complete	5.98	37.71	Salihorsk District, Belarus	Potash salt dump	E
Pm	5-3	GCA_009911775	Complete	5.17	38.30	Shangluo, China	Farmland	P
Pm	KNU-01	GCA_006385935	Complete	5.71	37.88	South Korea	Soil	E
Pm	IGA-FME-1	GCA_015643545	Complete	5.15	38.20	Lishu, Jilin, China	Bulk soil of maize	P
Pm	H2	GCA_017352315	Complete	6.41	37.49	Turkey: Tuz Golu	Hypersaline environment	E
Pm	YC4-R4	GCA_003072605	Complete	5.43	38.10	Fujian, China	Rhizosphere of *Spartina anglica* (Hubb.) grow in salty-soil	P
Pm	JX285	GCA_002009195	Complete	5.61	37.90	Nanchang, China	Rhizosphere *Camellia oleifera*	P
Pm	HGS7	GCA_017798265	Complete	5.27	38.14	Chongqing, China	Mulberry	P
Pm	A	GCA_009497655	Complete	5.24	38.15	NA	Plant	P
Pm	CDC 2008724129	GCA_017086545	Complete	5.61	38.08	Rhode Island, USA	*Homo sapiens*	C
Pm	NCT-2	GCA_000334875	Complete	5.88	37.80	China	Secondary salinization soil of greenhouse	E
Pm	DSM 319	GCA_000025805	Complete	5.10	38.10	NA	NA	I
Pm	Q3	GCA_001050455	Complete	5.23	38.27	Hunan, China	Endosphere of tobacco grown in quinclorac contaminated soil	P
Pm	QM B1551	GCA_000025825	Complete	5.52	37.97	NA	NA	I
Pm	WSH-002	GCA_000225265	Complete	5.08	38.15	NA	NA	I
Pm	22-2^{R}	GCA_009935415	Scaffold	5.60	37.60	China	Commercial probiotic	I
Pa	B8W22^{T}	GCA_000956595	Contig	5.10	38.00	Hyderabad, India	NA	E
Pa	K13^{R}	GCA_002688605	Complete	5.25	38.14	Iksan, South Korea	Compost	I
Pf	NBRC 15715^{T}	GCA_001591565	Contig	3.91	37.60	NA	NA	O

Table S2. Co-up-DEGs GO ontologies enrichment.

Cluster	GO	Description	*p*-value
Biological Process	0009664	plant-type cell wall organization	4.9E-07
	0071555	cell wall organization	1.8E-06
	0045229	external encapsulating structure organization	7.2E-06
	0071669	plant-type cell wall organization or biogenesis	1.6E-04
	0071554	cell wall organization or biogenesis	2.2E-06
Molecular Function	0005199	structural constituent of cell wall	1.5E-08
	0016762	xyloglucan:xyloglucosyl transferase activity	3.2E-02
	0004601	peroxidase activity	1.0E-02
	0016684	oxidoreductase activity, acting on peroxide as acceptor	1.3E-02
	0016209	antioxidant activity	3.4E-02
	0046906	tetrapyrrole binding	3.3E-03
	0020037	heme binding	3.0E-02
Cellular Component	0005615	extracellular space	3.1E-02
	0005618	cell wall	5.9E-04
	0030312	external encapsulating structure	8.1E-04
	0005576	extracellular region	1.2E-02
	0043231	intracellular membrane-bounded organelle	1.1E-05
	0043227	membrane-bounded organelle	9.1E-06
	0005622	intracellular anatomical structure	7.9E-08
	0043229	intracellular organelle	5.5E-06
	0043226	organelle	4.7E-06

Table S3. Co-down-DEGs GO ontologies enrichment.

Cluster	GO	Description	*p*-value
Biological Process	1900366	negative regulation of defense response to insect	2.5E-02
	2000068	regulation of defense response to insect	4.2E-03
	2000022	regulation of jasmonic acid mediated signaling pathway	1.1E-06
	0071456	cellular response to hypoxia	6.4E-22
	0036294	cellular response to decreased oxygen levels	7.8E-22
	0071453	cellular response to oxygen levels	8.5E-22
	0001666	response to hypoxia	9.0E-24
	0036293	response to decreased oxygen levels	1.7E-23
	0070482	response to oxygen levels	2.1E-23
	0010200	response to chitin	7.4E-12
	0009404	toxin metabolic process	1.7E-02
	0009753	response to jasmonic acid	3.4E-17
	0070542	response to fatty acid	4.0E-17
	0010243	response to organonitrogen compound	3.7E-13
	0009751	response to salicylic acid	1.6E-06
	0009611	response to wounding	1.4E-14
	1901698	response to nitrogen compound	7.9E-14
	0014070	response to organic cyclic compound	1.8E-09
	0031347	regulation of defense response	1.6E-08
	0006979	response to oxidative stress	4.3E-06
	0019748	secondary metabolic process	2.9E-09
	0009414	response to water deprivation	2.2E-11
	0009620	response to fungus	1.5E-10
	0009415	response to water	2.4E-11
	0033554	cellular response to stress	6.4E-12
	0001101	response to acid chemical	5.2E-11
	0080134	regulation of response to stress	6.5E-08
	0006970	response to osmotic stress	5.0E-07
	0009651	response to salt stress	1.0E-03
	0050832	defense response to fungus	1.3E-03
	0070887	cellular response to chemical stimulus	1.3E-15
	0033993	response to lipid	7.0E-13
	1901700	response to oxygen-containing compound	7.3E-24
	0097305	response to alcohol	1.5E-06
	0010035	response to inorganic substance	5.4E-14
	0043207	response to external biotic stimulus	4.0E-13
	0051707	response to other organism	4.0E-13
	0009607	response to biotic stimulus	4.2E-13
	0044419	biological process involved in interspecies interaction between organisms	5.3E-13
	0048583	regulation of response to stimulus	4.1E-05
	0009737	response to abscisic acid	1.5E-03
	0009605	response to external stimulus	3.8E-14

	0009725	response to hormone	6.8E-11
	0009719	response to endogenous stimulus	1.1E-10
	0042221	response to chemical	2.2E-28
	1901701	cellular response to oxygen-containing compound	4.5E-03
	0010033	response to organic substance	2.3E-15
	0006950	response to stress	2.2E-26
	0098542	defense response to other organism	8.0E-07
	0009628	response to abiotic stimulus	1.9E-17
	0006952	defense response	6.8E-08
	0009617	response to bacterium	1.8E-02
	0071310	cellular response to organic substance	4.5E-03
	0051716	cellular response to stimulus	4.1E-12
	0006082	organic acid metabolic process	4.3E-04
	0043436	oxoacid metabolic process	3.5E-03
	0007154	cell communication	5.4E-04
	0007165	signal transduction	1.0E-02
	0023052	signaling	1.5E-02
	0050896	response to stimulus	2.6E-26
	0044281	small molecule metabolic process	1.6E-02
Molecular Function	0120091	jasmonic acid hydrolase	9.3E-03
	0016491	oxidoreductase activity	2.2E-02
Cellular Component	0032991	protein-containing complex	4.1E-02

Table S7. Genes associated with rhizosphere competence, plant growth-promoting and associated, salinity stress alleviation as well and oxygen and carbon resource limitation identified in *Priestia megaterium* BP01R2.

Category	Function	Gene name	Description (NCBI Protein, GenBank)	BP01R2 locus_tag (MGJ28_RS)
Rhizosphere competence	phosphate solubilization and mineralization	*gcd*	glucose dehydrogenase, PQQ-dependent	20270
		phoP	two-component response regulator PhoP	24715, 11185, 15795, 24765
		phoR	two-component sensor histidine kinase PhoR	10820, 24760, 15790
		phoA, phoB	alkaline phosphatase	06180
		phoD	alkaline phosphatase D	26445
		ppx	exopolyphosphatase Ppx	06650
	nitrogen assimilation and reduction	*narK, nasA*	nitrite extrusion protein	03965
		nasC	nitrate reductase, catalytic subunit	03960
		nasBD, nirB	nitrate reductase, electron transfer subunit	03955, 05855
		nasE, nirD	nitrite reductase [NAD(P)H], small subunit	05860
	siderophore synthesis/Fe-uptake	*na*	siderophore biosynthesis protein	21240, 21245, 21250, 21255, 21260, 21265
		na	polyketide synthase	07325
		yfmC	iron(III)-citrate import ABC transporter, iron(III)-citrate-binding protein	04445
		yfmD	iron(III)-citrate import ABC transporter, permease protein	04450
		yfmE	iron(III)-citrate import ABC transporter, permease protein	04455
Plant-associated	L-tryptophane, indole synthesis	*trpA*	tryptophan synthase, alpha subunit	22520
		trpB	tryptophan synthase, beta subunit	22525
		trpC	indole-3-glycerol-phosphate synthase	22535
		trpD	anthranilate phosphoribosyltransferase	22540, 16125
		trpE	anthranilate synthase component I	00470, 22545
		trpF	N-(5'-phosphoribosyl) anthranilate isomerase	22530
	auxin (indole-3-acetic acid) synthesis	*gatA, iaaH*	glutamyl-tRNA(Gln) and/or aspartyl-tRNA(Asn) amidotransferase, A subunit	01505, 05255, 17275

	auxin transport	*na*	auxin efflux carrier (AEC) family transporter	09980, 14190, 14240, 19315
	acetoin, butanediol synthesis	*alsD, budA, aldC*	alpha-acetolactate decarboxylase	04010
		alsS	acetolactate synthase, catabolic	04015, 08580
		ilvH, ilvN	acetolactate synthase, small subunit	24430
		ilvB, ilvG, ilvI	acetolactate synthase, large subunit, biosynthetic type	24435, 04830
		bdhA	2,3-butanediol dehydrogenase	12195, 25665, 11290, 15390, 11965, 09800
	flagellar assembly	*flgB*	flagellar basal-body rod protein FlgB	21900
		flgC	flagellar basal-body rod protein FlgC	21895
		flgD	flagellar hook assembly protein	21850
		flgE	flagellar hook protein FlgE	21845
		flgK	flagellar hook-associated protein FlgK	26530, 10685
		flgL	flagellar hook-associated protein FlgL	26525
		flgM	negative regulator of flagellin synthesis (anti-sigma-D factor)	26540
		flgN	conserved hypothetical protein	26535
		flhA	flagellar biosynthesis protein FlhA	21790
		flhB	flagellar biosynthetic protein FlhB	21795
		hag, fliC	flagellin	05595
		fliD	flagellar hook-associated protein FliD	26480
		fliE	flagellar hook-basal body complex protein FliE	21890
		fliF	flagellar M-ring protein FliF	21885
		fliG	flagellar motor switch protein FliG	21880
		fliH	flagellar assembly protein FliH	21875
		fliI	flagellum-specific ATP synthase	21870
		fliJ	flagellar export protein FliJ	21865
		fliK	flagellar hook-length control protein	21855
		fliM	flagellar motor switch protein FliM	21830
		fliN, fliY	flagellar motor switch protein FliN	21825
		fliP	flagellar biosynthetic protein FliP	21810
		fliQ	flagellar biosynthetic protein FliQ	21805
		fliR	flagellar biosynthetic protein FliR	21800

		fliT	flagellar assembly protein FliT	26470
		fliS	flagellar protein FliS	26475, 26570
		motA	chemotaxis protein MotA	10425
		motB	chemotaxis protein MotB	10430
	bacterial chemotaxis	*cheA*	chemotactic two-component sensor histidine kinase	21770
		cheB	chemotactic two-component response regulator-glutamate methylesterase	21775
		cheD	chemoreceptor glutamine deamidase CheD	21760
		cheR	chemotaxis protein methyltransferase	07775, 22560
		cheY	chemotaxis protein CheY	04760, 21820
		cheW	chemotactic signal transduction protein	21765
Salinity stress alleviation	superoxide dismutase	*sodACF*	superoxide dismutase	10920, 23510, 25700, 14750
	catalase	*cat*	catalase	16360, 27180, 20625, 17200, 17205
	spermidine synthesis	*speA*	arginine decarboxylase	06820, 00240
		speB	agmatinase	11530, 26925, 04410
		speH, speD, AMD1	S-adenosylmethionine decarboxylase	24730, 15765
		speE, SRM	spermidine synthase	26930, 03970
Oxygen and carbon resource limitation	anaerobic respiration	*ldh*	L-lactate dehydrogenase	02650
		alsS	acetolactate synthase AlsS	04015
		budA	acetolactate decarboxylase	04010
		pta	phosphate acetyltransferase	26985
		na	acetate kinase	24900
		ar	2,3-butanediol dehydrogenase	09800
		acoABC	acetoin dehydrogenas	09775, 09780, 09785
		pdh	pyruvate dehydrogenase	06785, 06790, 06795, 03690, 06800
		acsA	acetyl-CoA synthetas	12695, 24990
	anaerobic regulatory	*resA*	thiol-disulfide oxidoreductase ResA	22785
		na	cytochrome c biogenesis protein ResB	22780

		ccsB	c-type cytochrome biogenesis protein CcsB	22775
		na	response regulator transcription factor	22770
		na	ATP-binding protein	22765
		fnr	Fnr family transcriptional regulator	03420
	reductive glycine (rGly) pathway	*fdhF*	formate dehydrogenase subunit alpha	12430, 12505, 26155
		fdhD	formate dehydrogenase accessory sulfurtransferase FdhD	26145, 12455
		focA	formate transporter	05870, 12745
		ftl	formate-tetrahydrofolate (THF) ligase	10120
		folD	methenyltetrahydrofolate cyclohydrolase/methylenetetrahydrofolate dehydrogenase (NADP+)	10735
		gcvPA	glycine dehydrogenase (glycine cleavage system P1 protein)	23360
		gcvPB	glycine dehydrogenase (glycine cleavage system P2 protein)	23355
		gcvH	glycine cleavage system protein H-protein	25960
		gcvT	glycine cleavage system T-protein	23365
		shmt	serine hydroxymethyltransferase	26770
		sdaAB	L-serine ammonia-lyase, iron-sulfur-dependent subunit beta	22055
		sdaAA	L-serine ammonia-lyase, iron-sulfur-dependent, subunit alpha	22050
		na	D-serine ammonia-lyase	10635
	reductive tricarboxylic acid (rTCA) pathway	*pyc*	pyruvate carboxylase	06900
		ppc	phosphoenolpyruvate carboxylase	04135
		mdh	malate dehydrogenase	24775
		fum	fumarate hydratase	01970, 11865
		sdhABC	succinate dehydrogenase/fumarate reductase	24550, 24555, 24560
		sucCD	succinate-CoA ligase	21940, 21945
		korAB	2-oxoglutarate ferredoxin oxidoreductase	21490, 21485
		icd	NADP-dependent isocitrate dehydrogenase	24780
		acnA	aconitate hydratase AcnA	13820
		cs	citrate synthase CS	16565, 24785

	ferredoxin	*fer*	ferredoxin	12115, 17790, 22745, 04525, 12240
