# Supplementary material for: A cyclic dipeptide for salinity stress alleviation and the trophic flexibility of endophyte provide insights into saltmarsh plant–microbe interactions
Source: ISME Commun. 2024 Mar 26;4(1):ycae041. doi: 10.1093/ismeco/ycae041 (PMC11070113; doi:10.1093/ismeco/ycae041)
Supplement: Fig_S1toS22_ycae041 [file fig_s1tos22_ycae041.pdf]

ISME Communications Supplementary Figures

Article title:

A cyclic dipeptide for salinity stress alleviation and the trophic flexibility of an endophyte reveal niches in salt marsh plant-microbe interactions

Authors:

Shih-Hsun Walter Hung[1,2,3], Pin-Hsien Yeh[1], Tsai-Ching Huang[1], Shao-Yu Huang[1], I-Chen Wu[1], Chia-Ho Liu[1], Yu-Hsi Lin[1], Pei-Ru Chien[1], Fan-Chen Huang[1], Ying-Ning Ho[4,5], Chih-Horng Kuo[2,6], Hau-Hsuan Hwang[1,3,7], En-Pei Isabel Chiang[3,7,8] and Chieh-Chen Huang[1,3,7]*

Affiliations:

1 Department of Life Sciences, National Chung Hsing University, Taichung 402202, Taiwan

2 Institute of Plant and Microbial Biology, Academia Sinica, Taipei 115201, Taiwan

3 Advanced Plant and Food Crop Biotechnology Center, National Chung Hsing University, Taichung 402202, Taiwan

4 Institute of Marine Biology, College of Life Science, National Taiwan Ocean University, Keelung 202301, Taiwan

5 Center of Excellence for the Oceans, National Taiwan Ocean University, Keelung 202301, Taiwan

6 Biotechnology Center, National Chung Hsing University, Taichung 402202, Taiwan

7 Innovation and Development Center of Sustainable Agriculture, National Chung Hsing University, Taichung 402202, Taiwan

8 Department of Food Science and Biotechnology, National Chung Hsing University, Taichung 402202, Taiwan

*Corresponding authors: Chieh-Chen Huang, cchuang@dragon.nchu.edu.tw

Supplementary Figures

Fig. S1 Volcano plot of differential expression genes (DEGs).

Fig. S2 Gene set enrichment analysis (GSEA) of DEGs (BP01R2 vs CK) within biological process GO ontologies.

Fig. S3 Gene set enrichment analysis (GSEA) of DEGs (BP01R2 vs CK) within molecular functions GO ontologies.

Fig. S4 Gene set enrichment analysis (GSEA) of DEGs (BP01R2 vs CK) within KEGG pathways.

Fig. S5 Expression pattern of DEGs (BP01R2 vs CK) in KEGG glycolysis and gluconeogenesis pathway.

Fig. S6 Expression pattern of DEGs (BP01R2 vs CK) in KEGG oxidative phosphorylation pathway.

Fig. S7 Expression pattern of DEGs (BP01R2 vs CK) in KEGG plant hormone signal transduction pathway.

Fig. S8 Gene set enrichment analysis (GSEA) of DEGs (BP01R2_NaCl vs CK_NaCl) within biological process GO ontologies.

Fig. S9 Gene set enrichment analysis (GSEA) of DEGs (BP01R2_NaCl vs CK_NaCl) within KEGG pathways.

Fig. S10 Expression pattern of DEGs (BP01R2_NaCl vs CK_NaCl) in KEGG plant hormone signal transduction pathway.

Fig. S11 Expression pattern of DEGs (BP01R2_NaCl vs CK_NaCl) in MAPK signaling pathway.

Fig. S12 Expression pattern of DEGs (BP01R2_NaCl vs CK_NaCl) in ribosome pathway.

Fig. S13 Expression pattern of DEGs (BP01R2_NaCl vs CK_NaCl) in KEGG glutathione metabolism pathway.

Fig. S14 Expression pattern of DEGs (CK_NaCl compared CK) in KEGG plant hormone signal transduction pathway.

Fig. S15 Expression pattern of DEGs (CK_NaCl compared CK) in KEGG MAPK signaling pathway.

Fig. S16 Expression pattern of DEGs (CK_NaCl compared CK) in KEGG ribosome pathway.

Fig. S17 Expression pattern of DEGs (CK_NaCl compared CK) in glutathione metabolism pathway.

Fig. S18 IAA production evaluation of BP01R2.

Fig. S19 Genome map of *Priestia megaterium* BP01R2 chromosome.

Fig. S20 ABC transporters identified within the genome of BP01R2.

Fig. S21 Two-component systems identified within the genome of BP01R2.

Fig. S22 The multiple sequence alignment analysis of oligoribonuclease NrnB among *Priestia megaterium* and its relatives.

Supplementary Figures

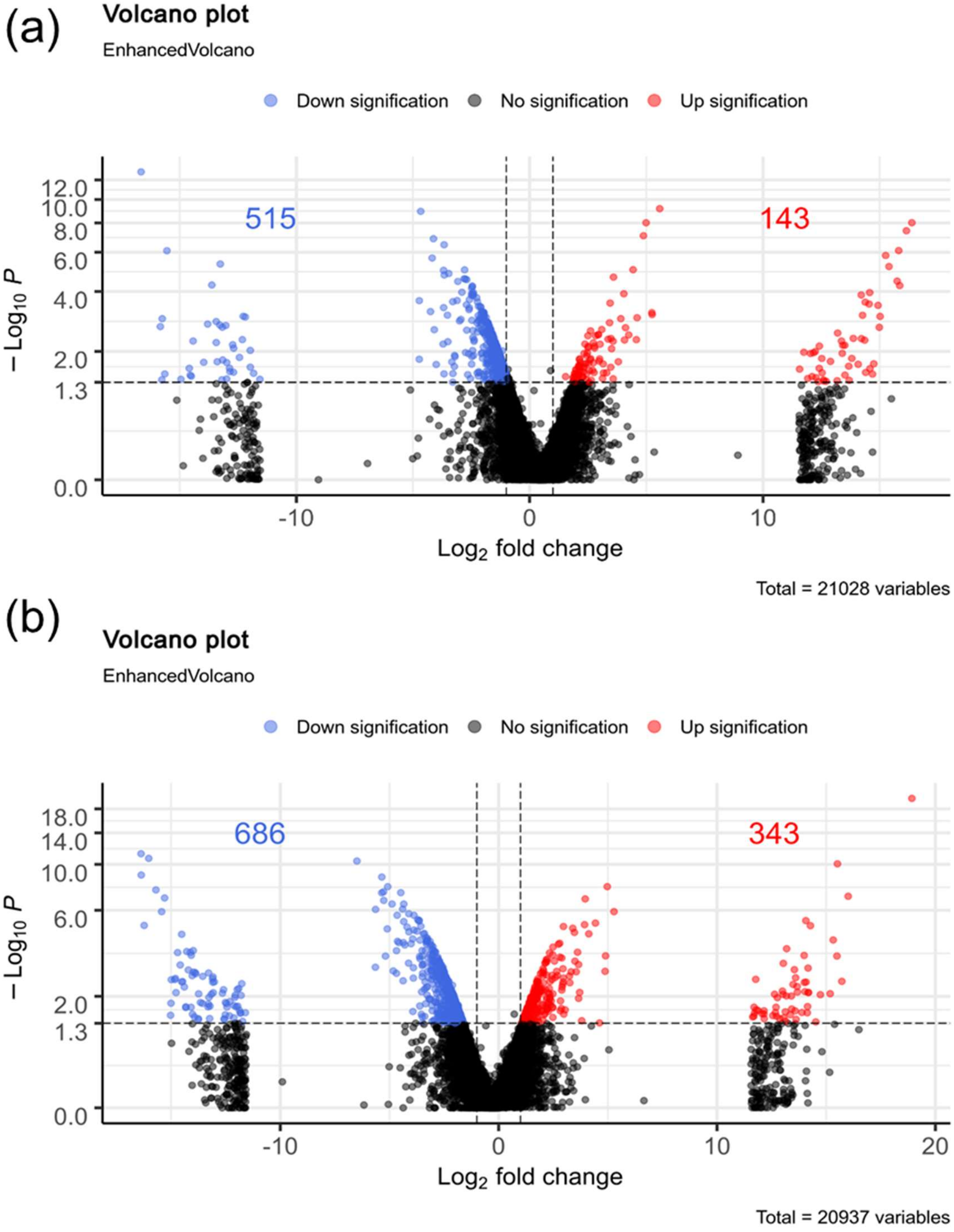

Fig. S1 Volcano plot of differential expression genes (DEGs). (a) BP01R2 vs CK. **(b)** BP01R2_NaCl vs CK_NaCl.

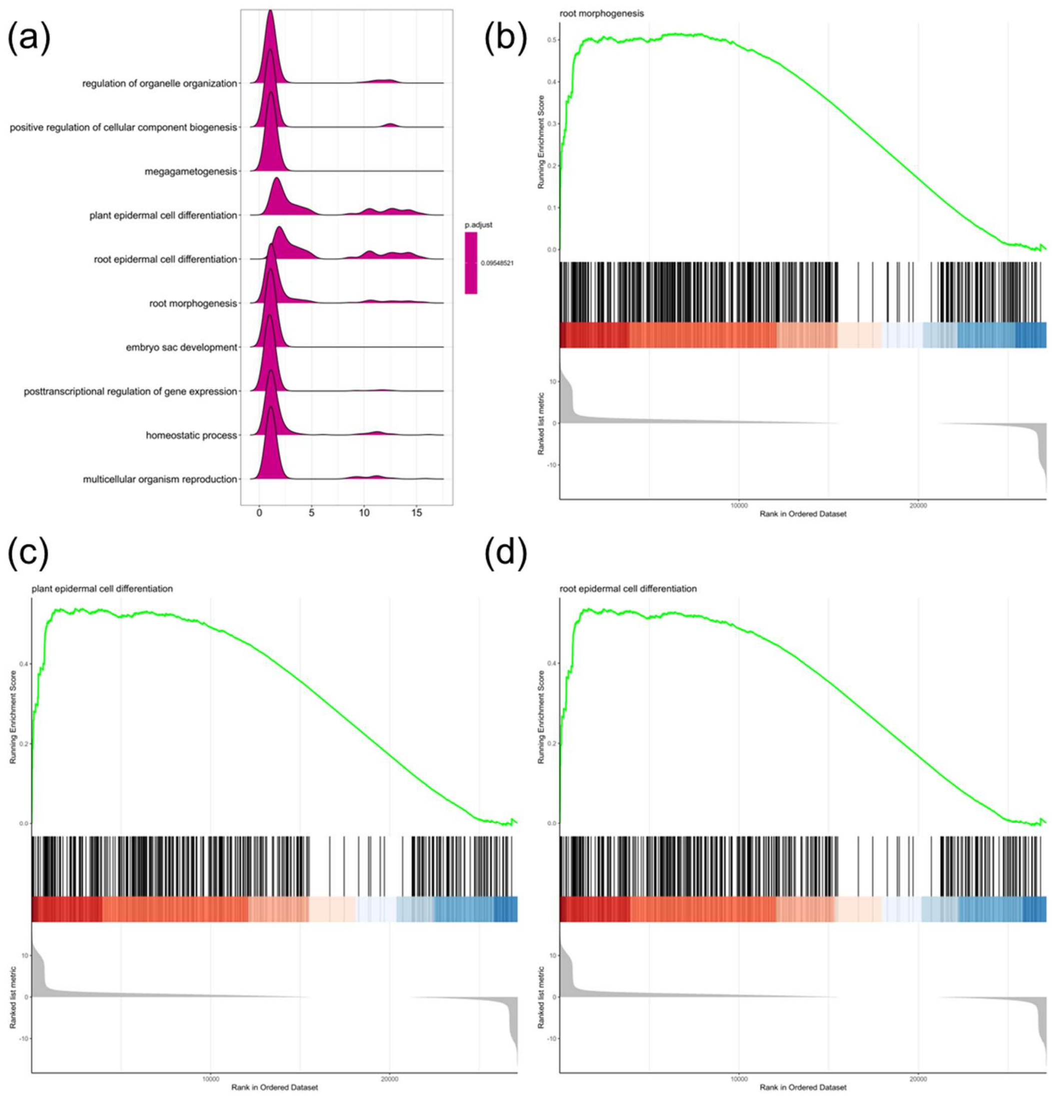

Fig. S2 Gene set enrichment analysis (GSEA) of DEGs (BP01R2 vs CK) within biological process GO ontologies. (a) Ridgeline plot for expression distribution. GSEA score plots of **(b)** root morphogenesis, **(c)** plant epidermal cell differentiation and **(d)** root epidermal cell differentiation.

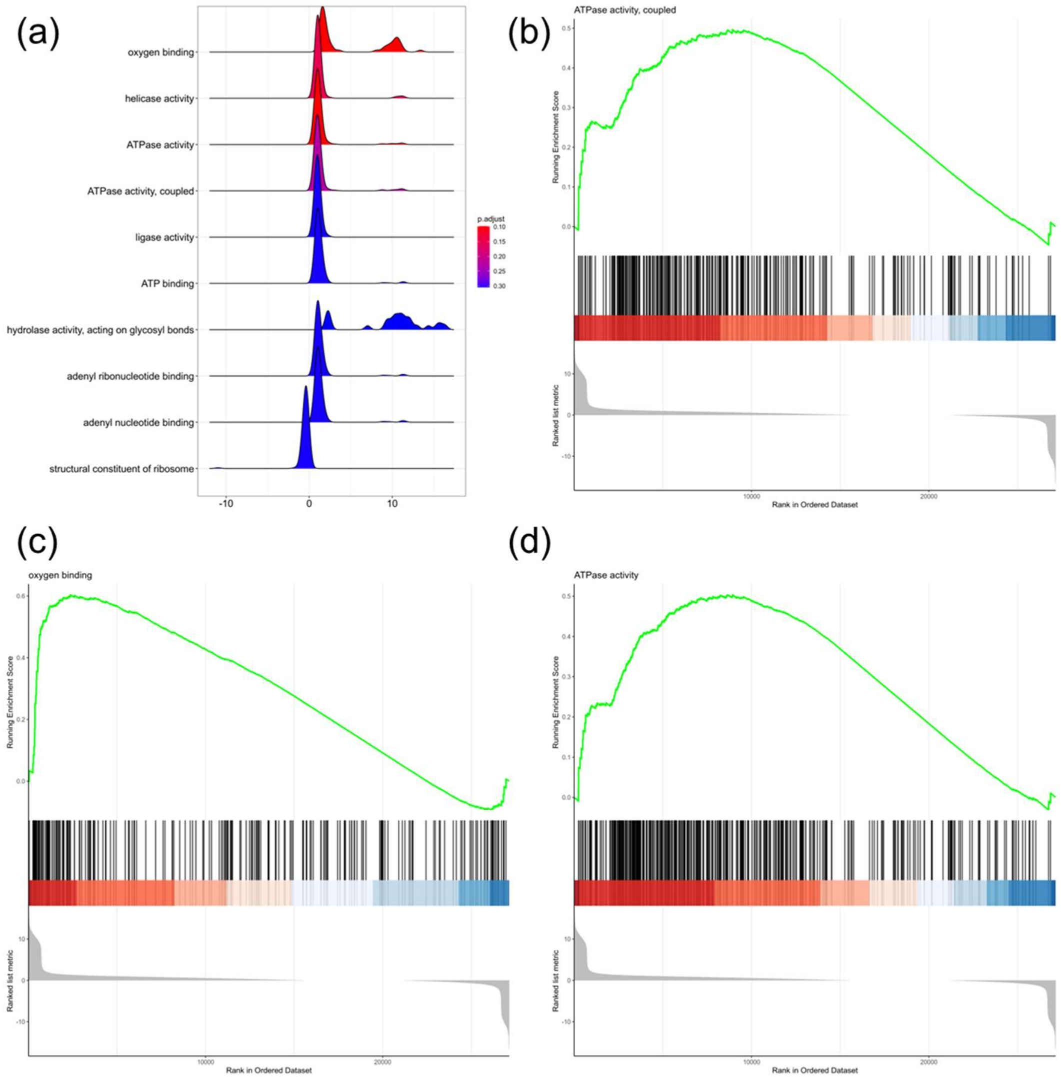

Fig. S3 Gene set enrichment analysis (GSEA) of DEGs (BP01R2 vs CK) within molecular functions GO ontologies. (a) Ridgeline plot for expression distribution. GSEA score plots of **(b)** ATPase activity, coupled, **(c)** oxygen binding and **(d)** ATPase activity.

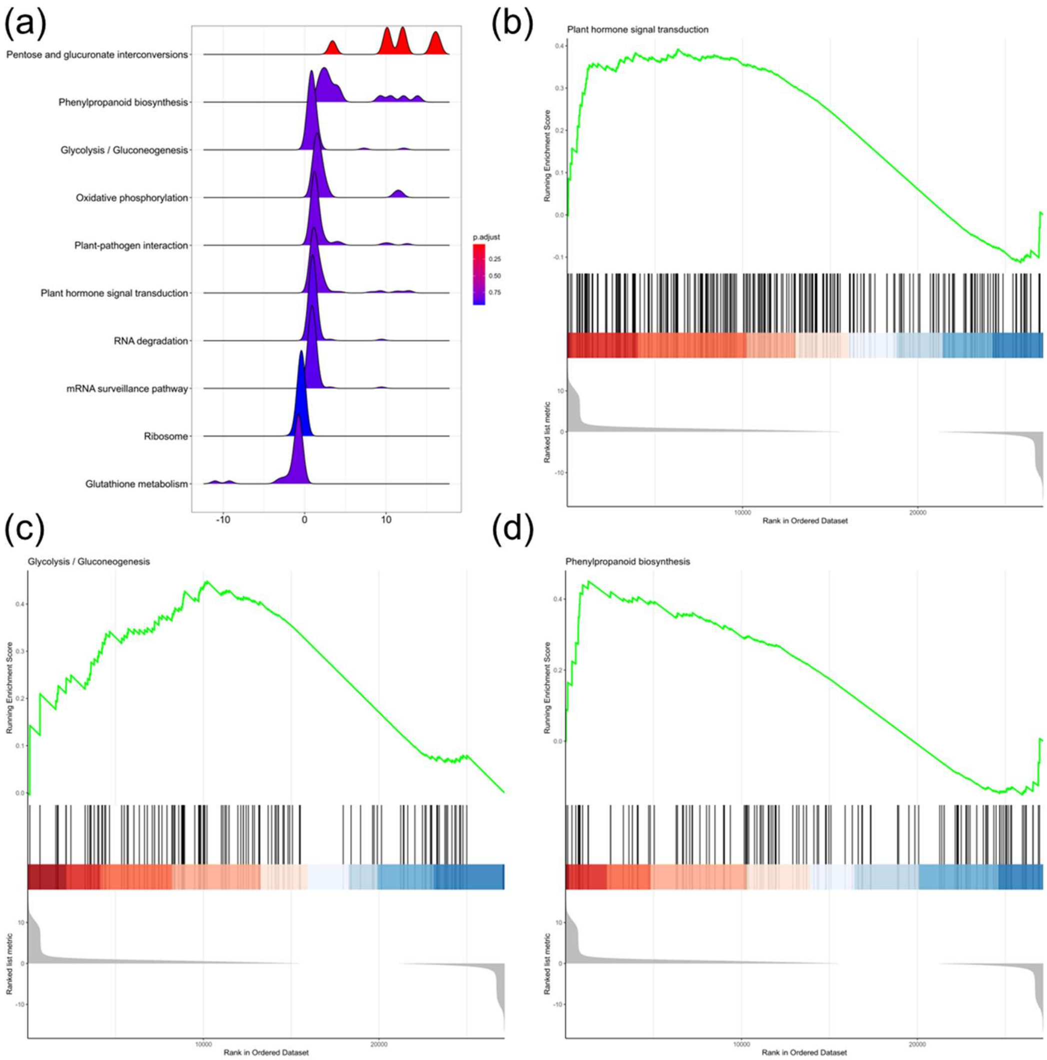

Fig. S4 Gene set enrichment analysis (GSEA) of DEGs (BP01R2 vs CK) within KEGG pathways. (a) Ridgeline plot for expression distribution. GSEA score plots of **(b)** plant hormone signal transduction, **(c)** glycolysis/gluconeogenesis and **(d)** phenylpropanoid biosynthesis.

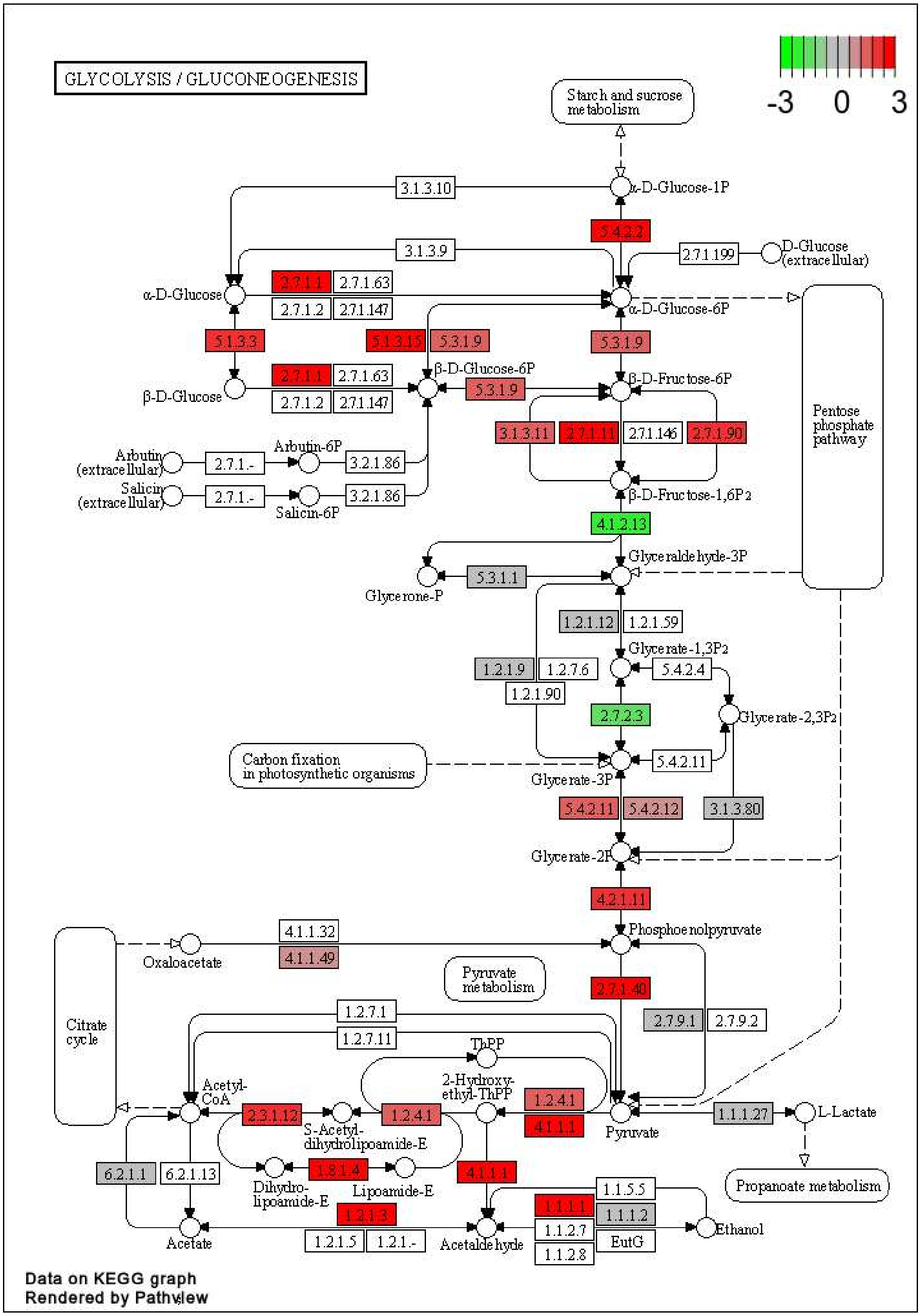

Fig. S5 Expression pattern of DEGs (BP01R2 vs CK) in KEGG glycolysis and gluconeogenesis pathway.

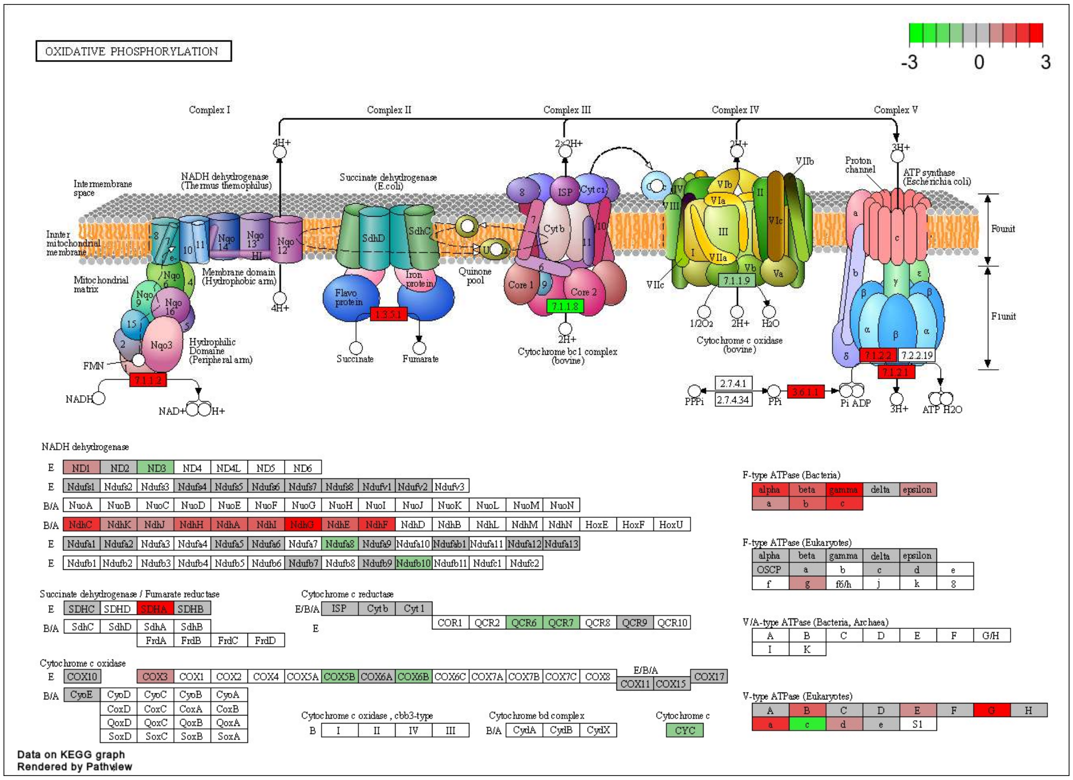

Fig. S6 Expression pattern of DEGs (BP01R2 vs CK) in KEGG oxidative phosphorylation pathway.

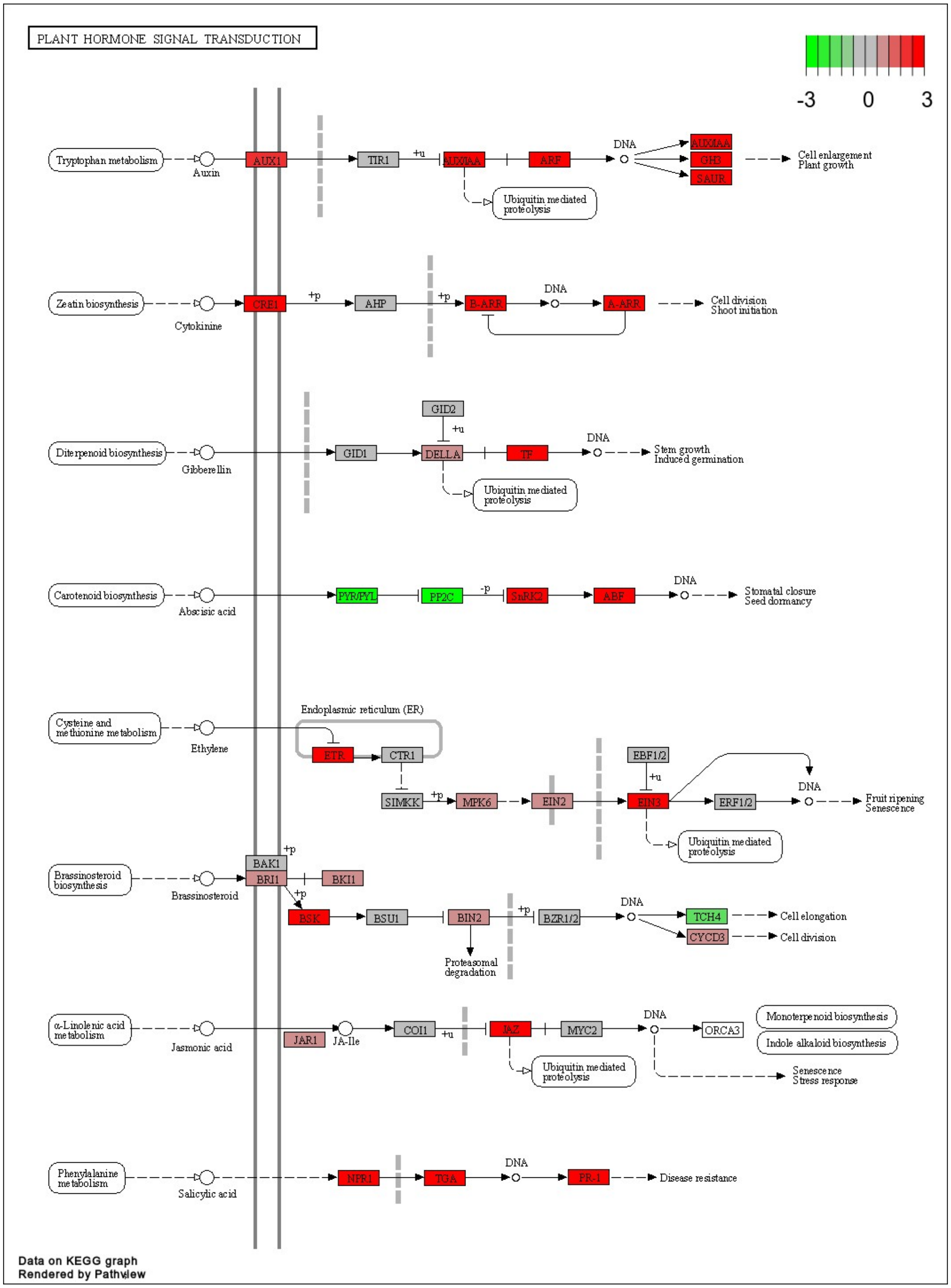

Fig. S7 Expression pattern of DEGs (BP01R2 vs CK) in KEGG plant hormone signal transduction pathway.

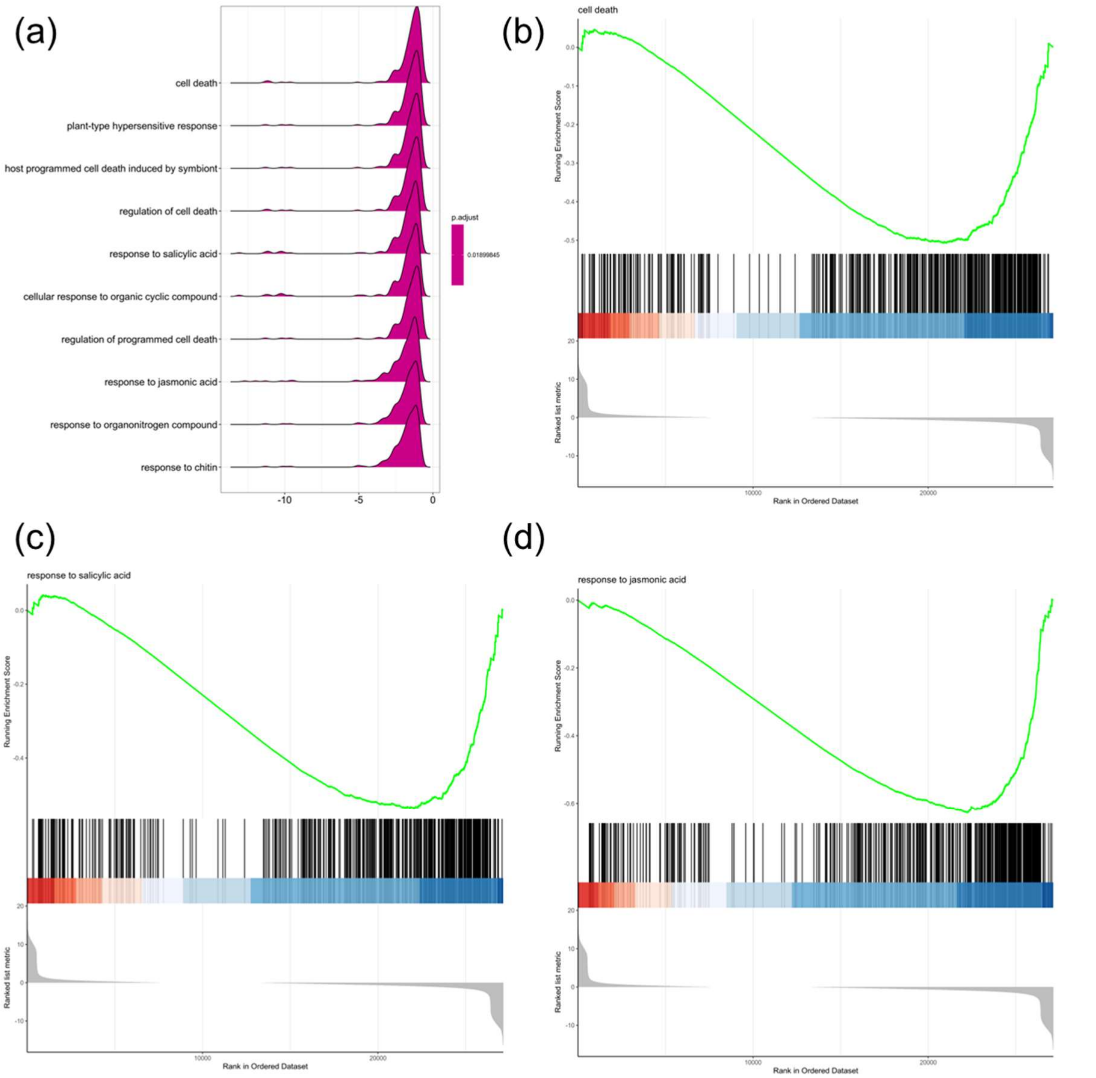

Fig. S8 Gene set enrichment analysis (GSEA) of DEGs (BP01R2_NaCl vs CK_NaCl) within biological process GO ontologies. (a) Ridgeline plot for expression distribution. GSEA score plots of **(b)** cell death, **(c)** response to salicylic acid and **(d)** response to jasmonic acid.

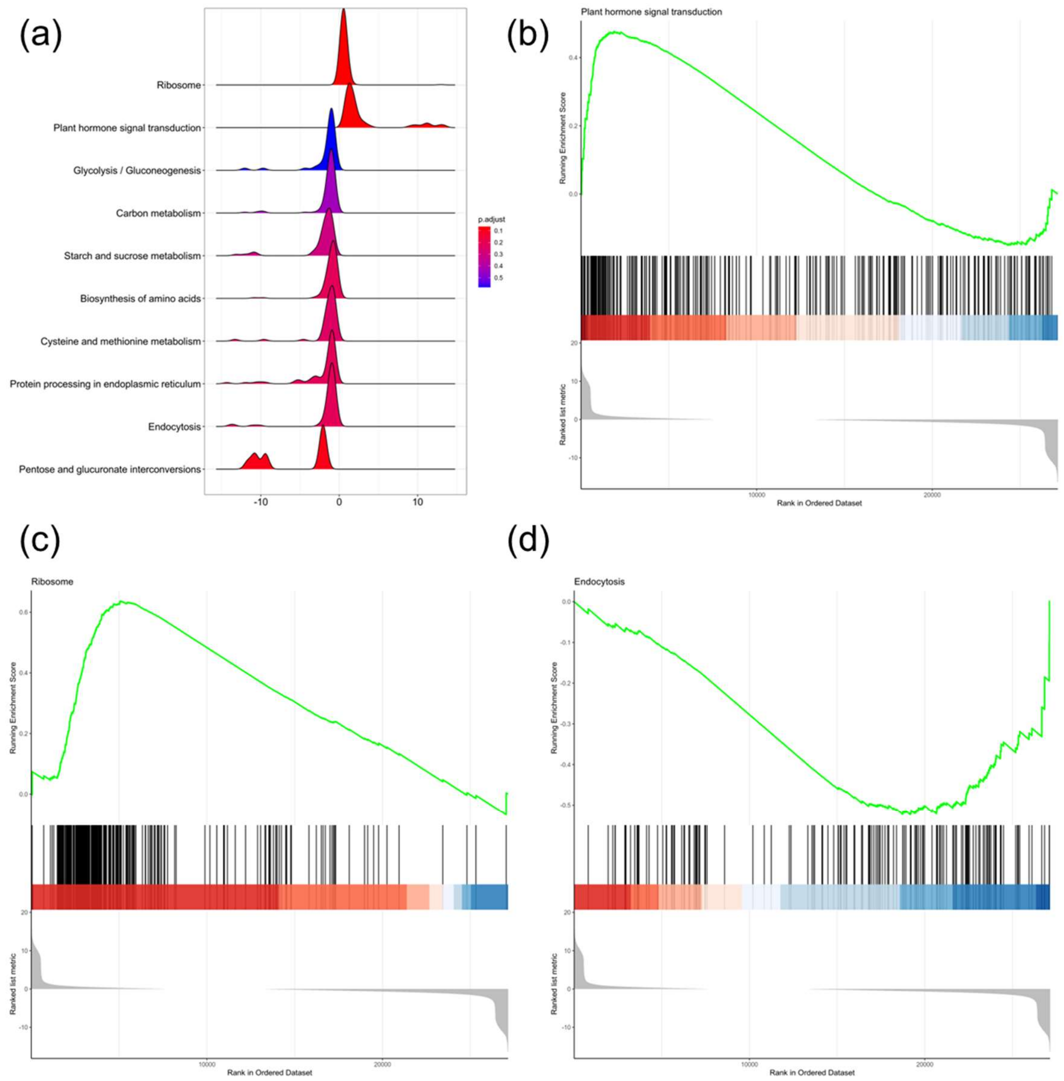

Fig. S9 Gene set enrichment analysis (GSEA) of DEGs (BP01R2_NaCl vs CK_NaCl) within KEGG pathways. (a) Ridgeline plot for expression distribution. GSEA score plots of **(b)** plant hormone signal transduction, **(c)** ribosome and **(d)** endocytosis.

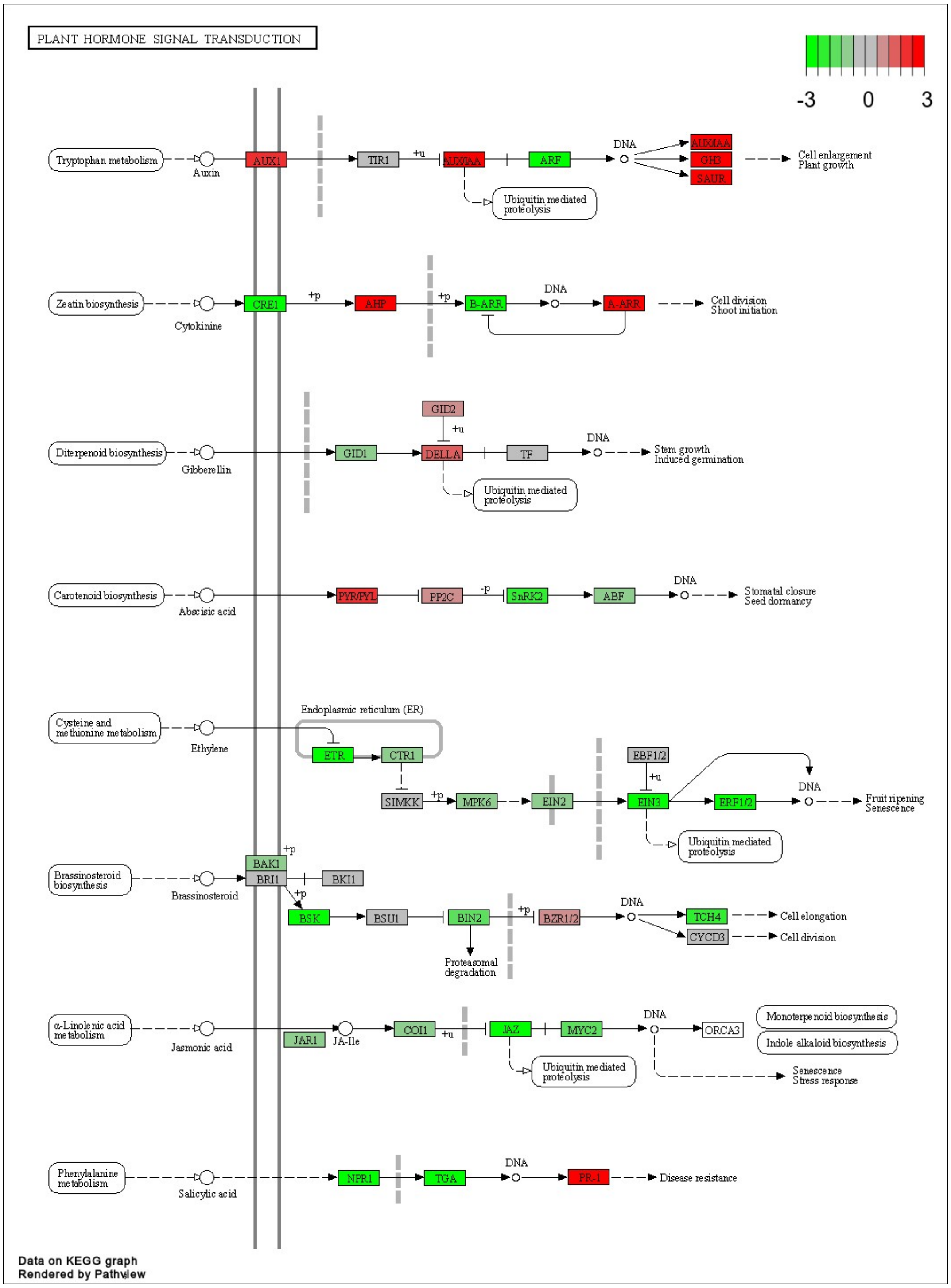

Fig. S10 Expression pattern of DEGs (BP01R2_NaCl vs CK_NaCl) in KEGG plant hormone signal transduction pathway.

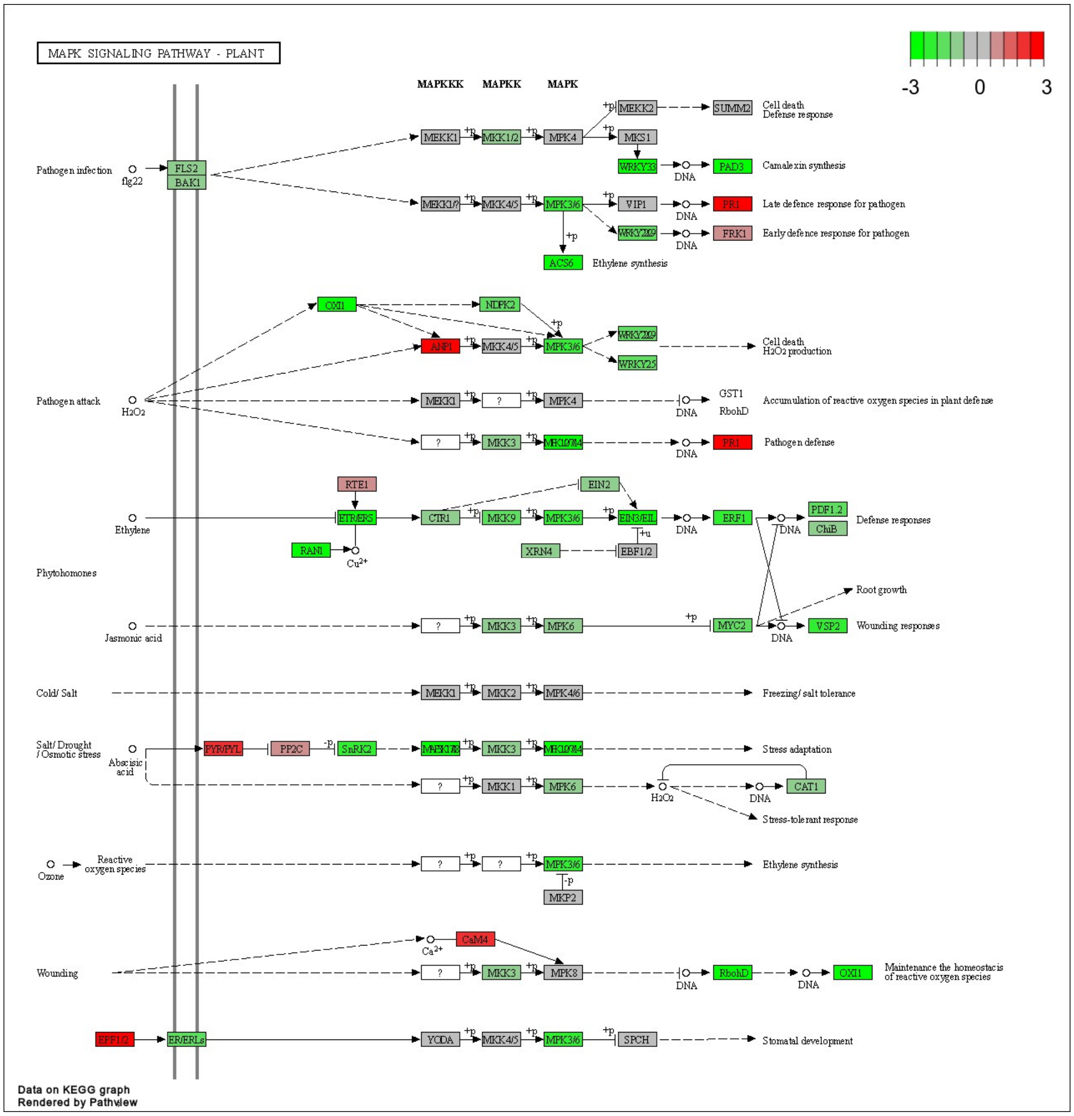

Fig. S11 Expression pattern of DEGs (BP01R2_NaCl vs CK_NaCl) in MAPK signaling pathway.

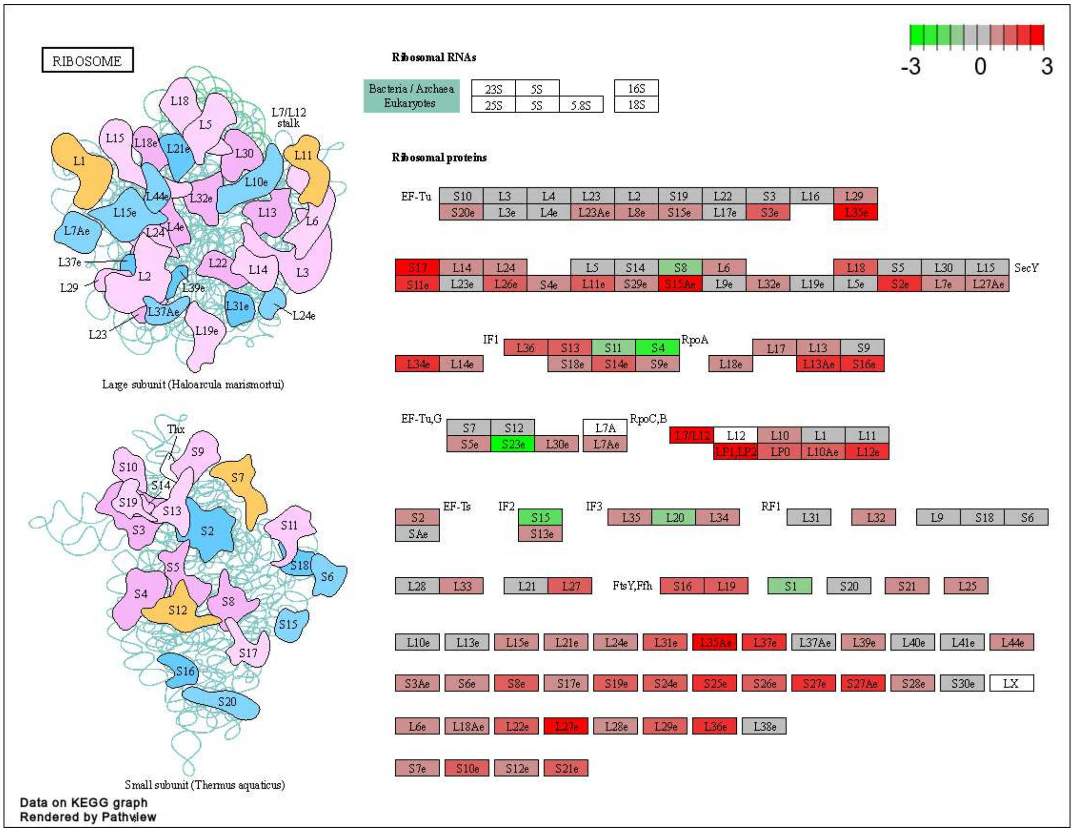

Fig. S12 Expression pattern of DEGs (BP01R2_NaCl vs CK_NaCl) in ribosome pathway.

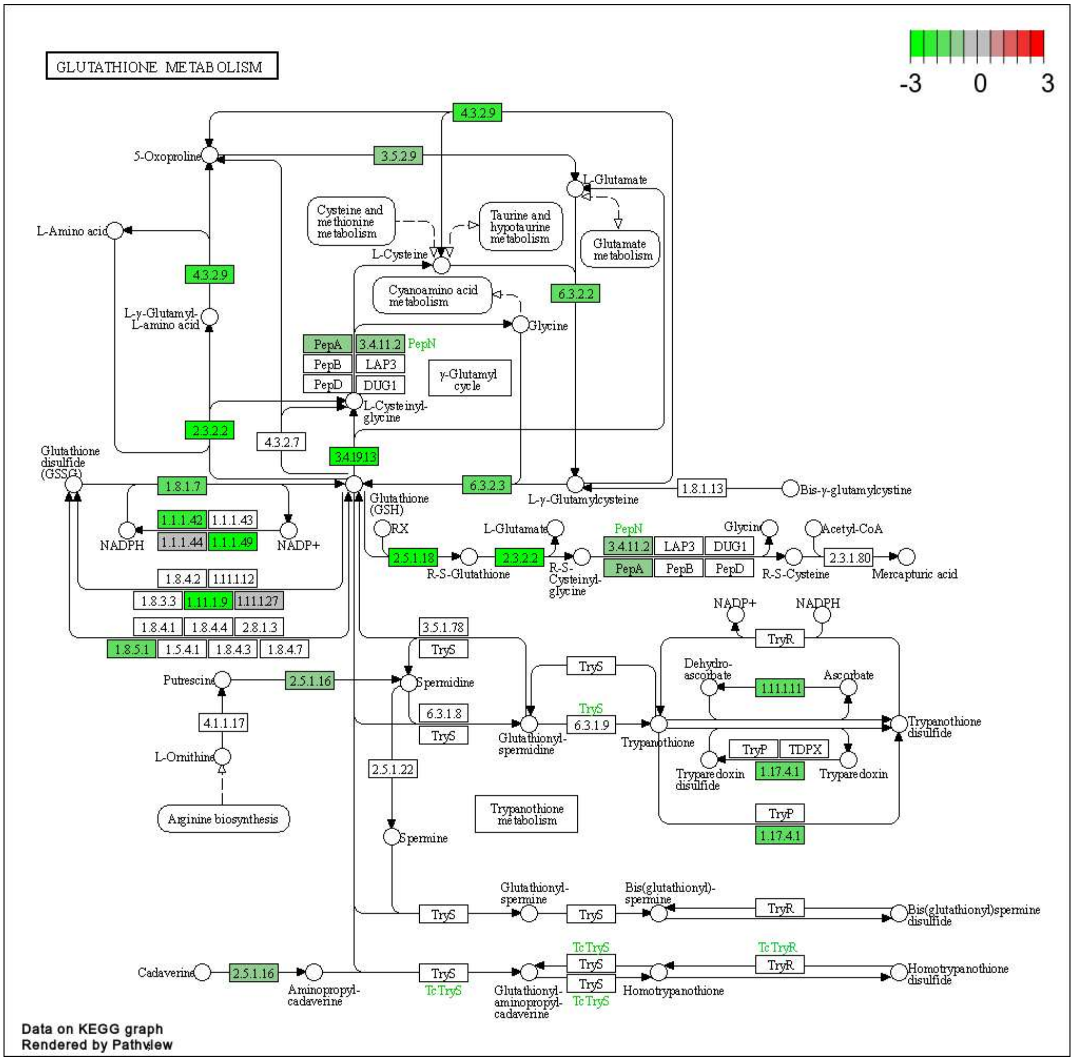

Fig. S13 Expression pattern of DEGs (BP01R2_NaCl vs CK_NaCl) in KEGG glutathione metabolism pathway.

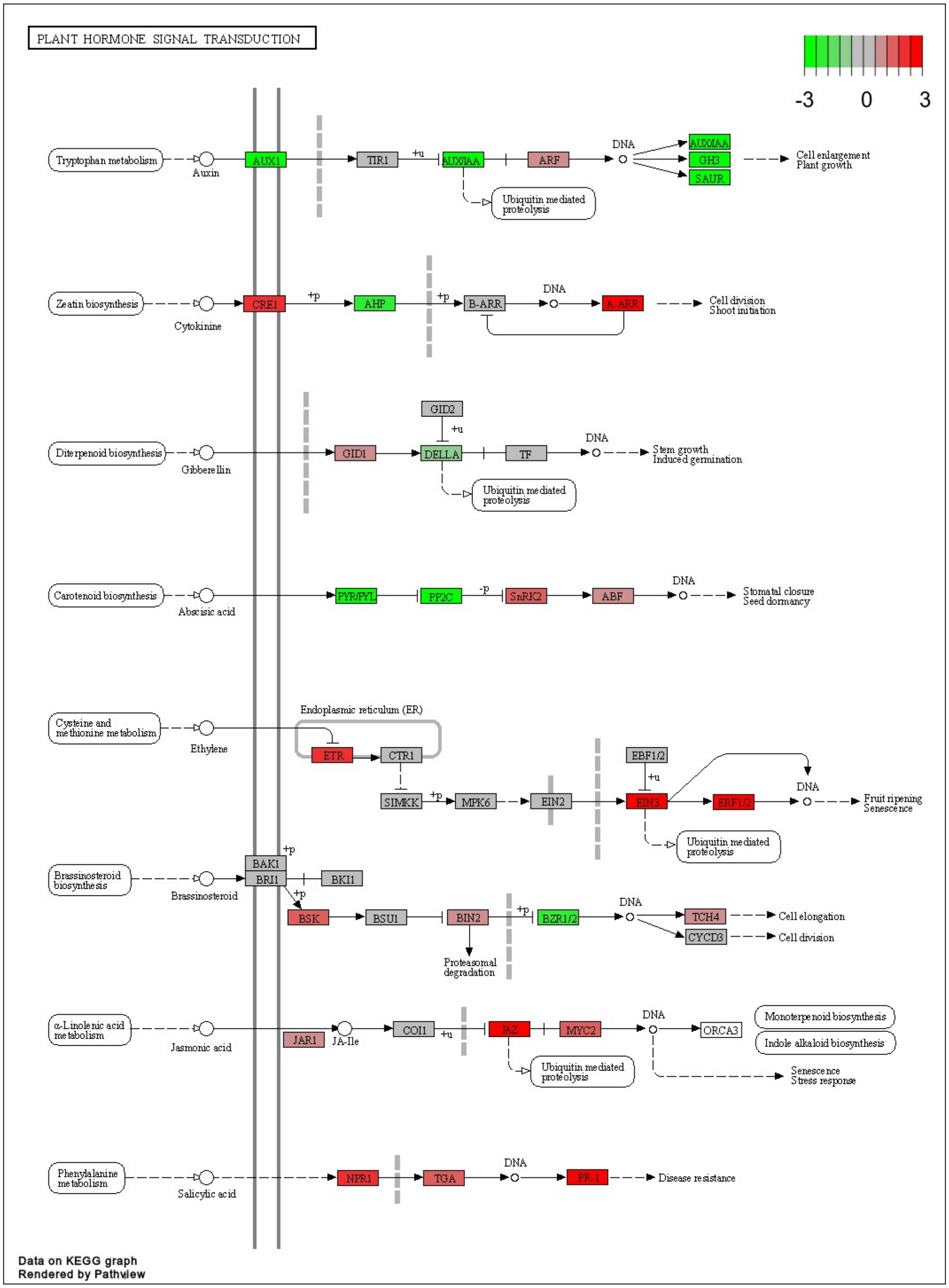

Fig. S14 Expression pattern of DEGs (CK_NaCl compared CK) in KEGG plant hormone signal transduction pathway.

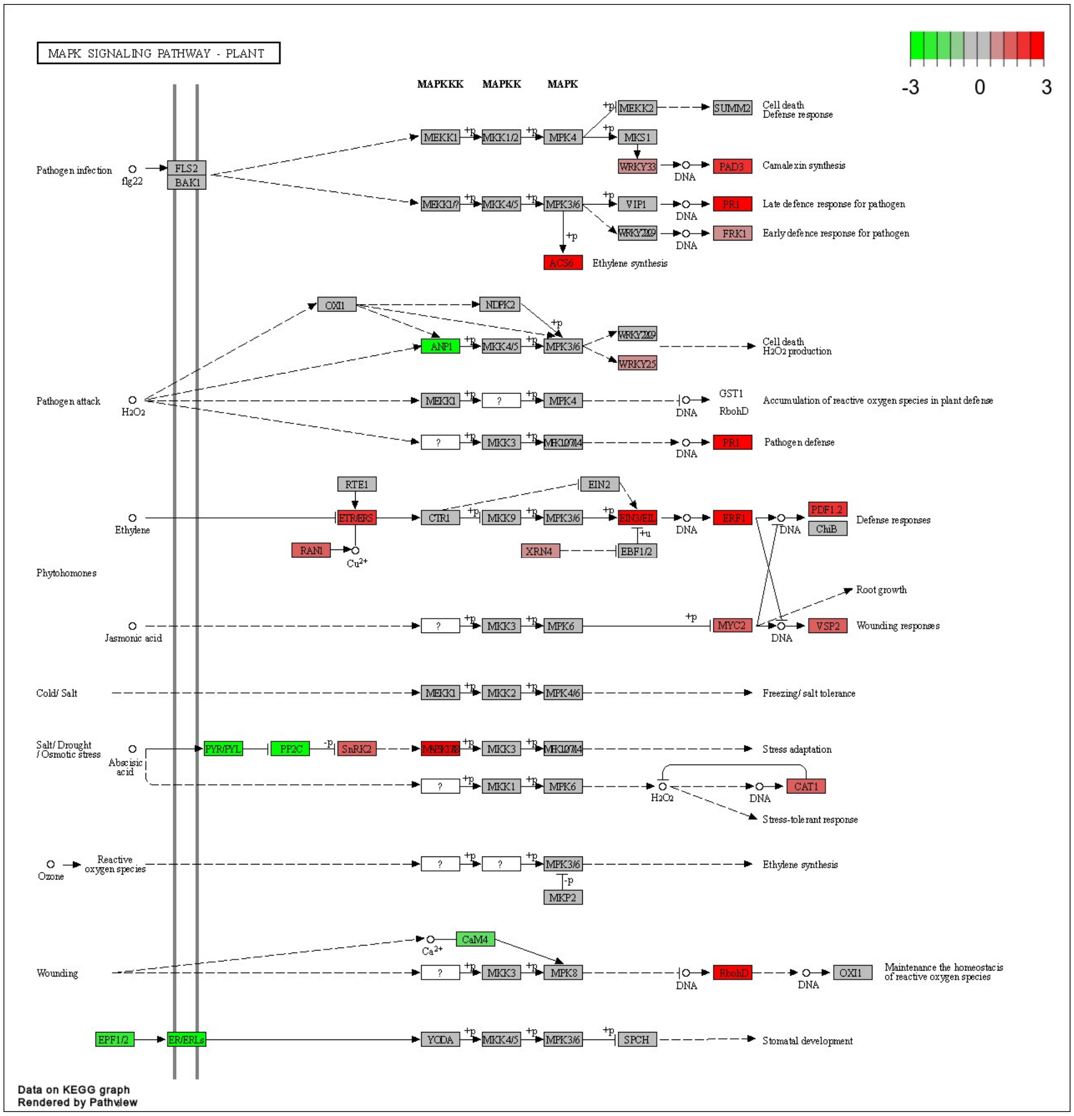

Fig. S15 Expression pattern of DEGs (CK_NaCl compared CK) in KEGG MAPK signaling pathway.

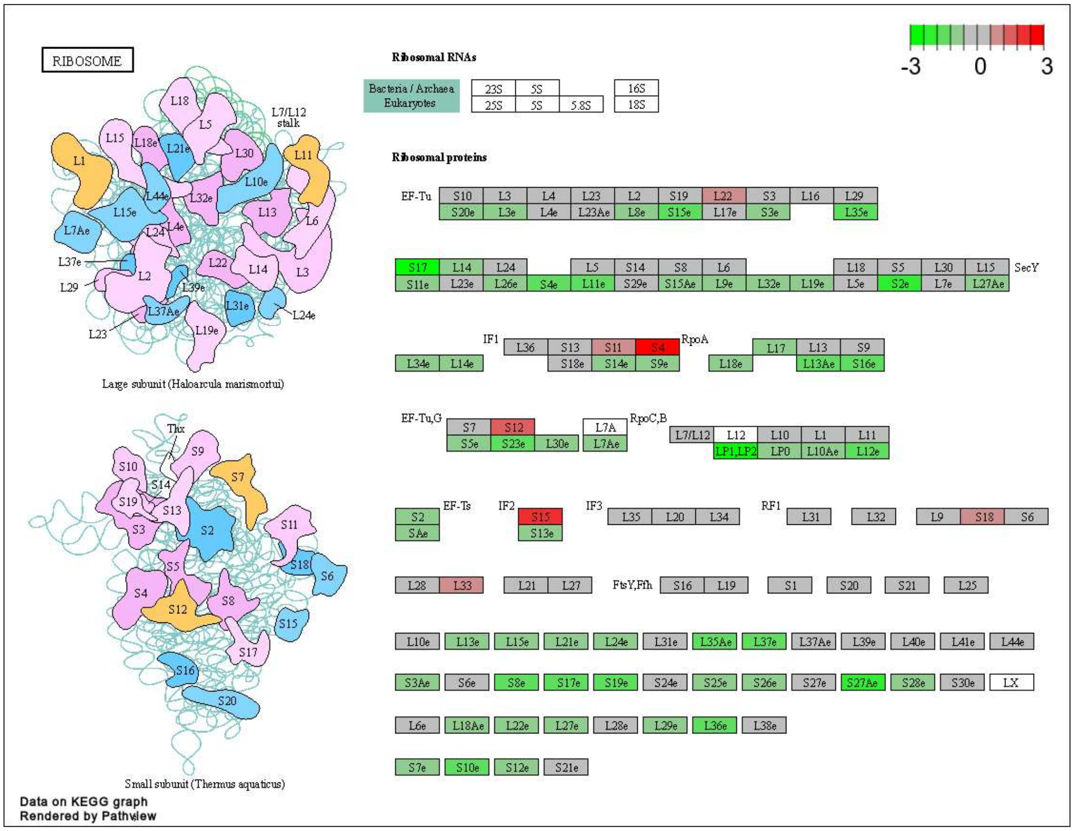

Fig. S16 Expression pattern of DEGs (CK_NaCl compared CK) in KEGG ribosome pathway.

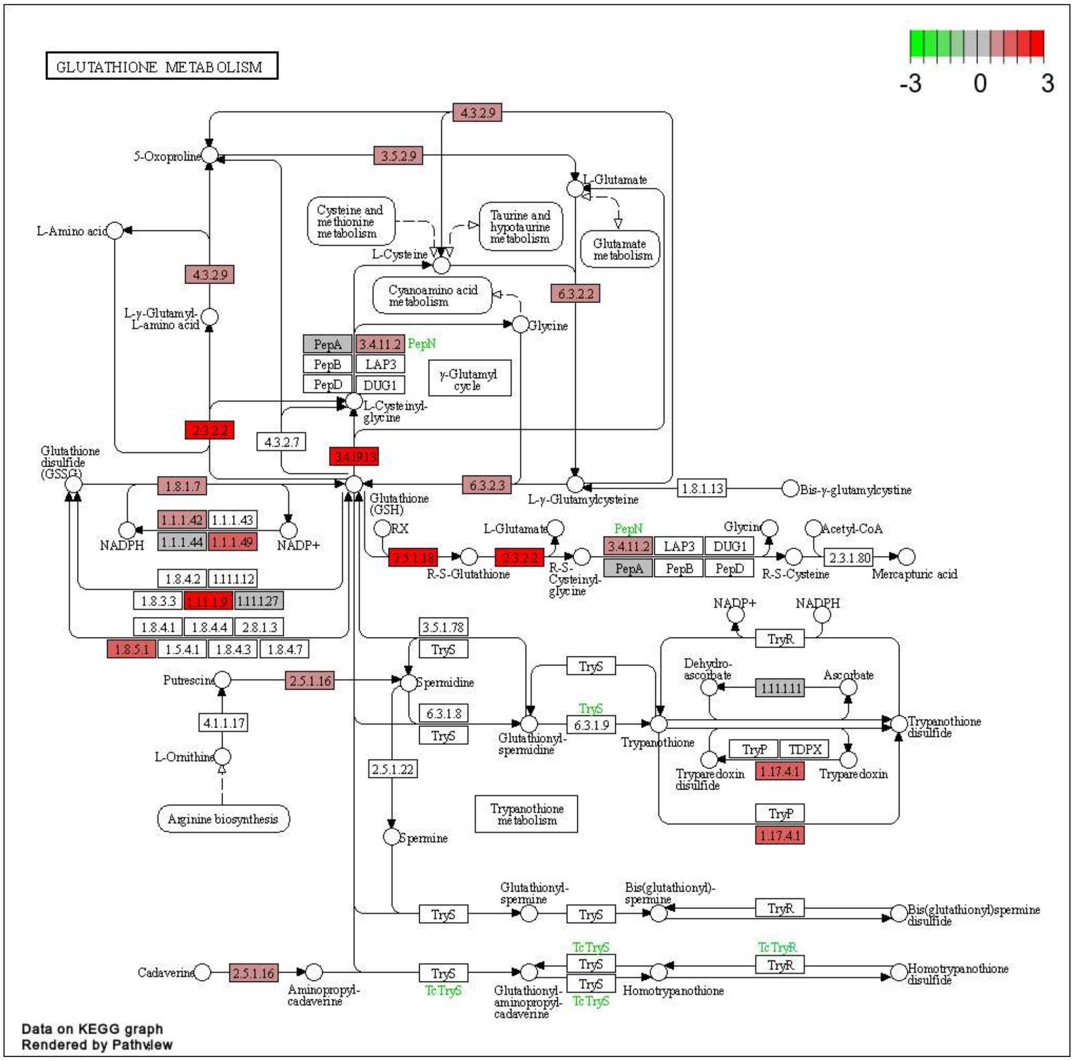

Fig. S17 Expression pattern of DEGs (CK_NaCl compared CK) in glutathione metabolism pathway.

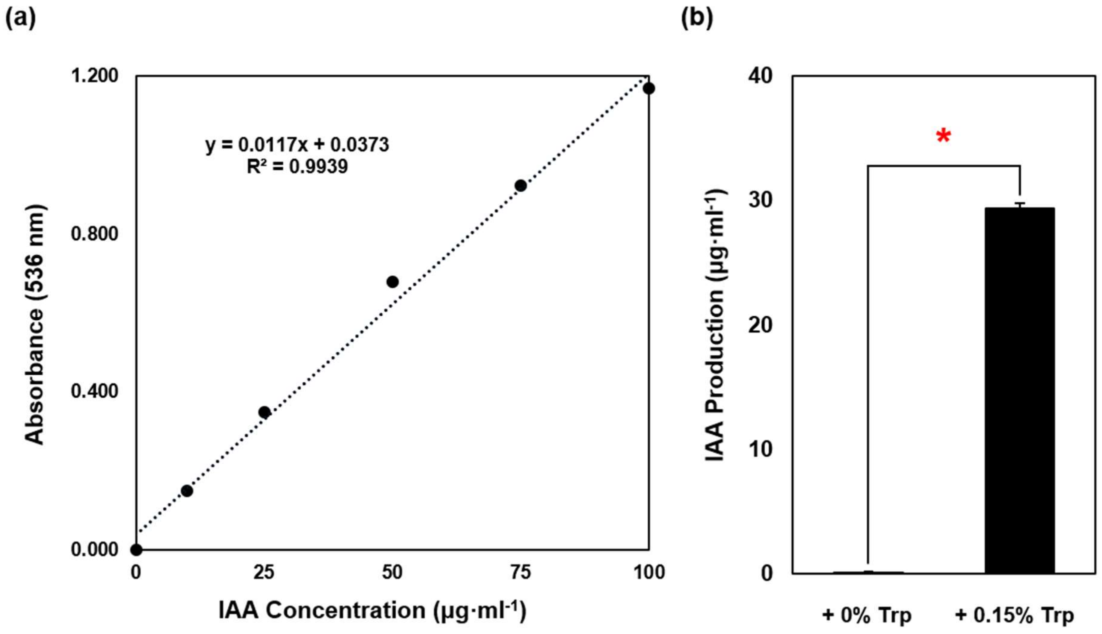

Fig. S18 IAA production evaluation of BP01R2. (a) Calibration curve of IAA. **(b)** IAA production was measured by Salkowski reagent methods after 24 hours of incubation with or without 0.15% L-Tryptophan (Trp) supplementation. Each sample was performed with at least three biological and three technical replications. *$p<0.05$.

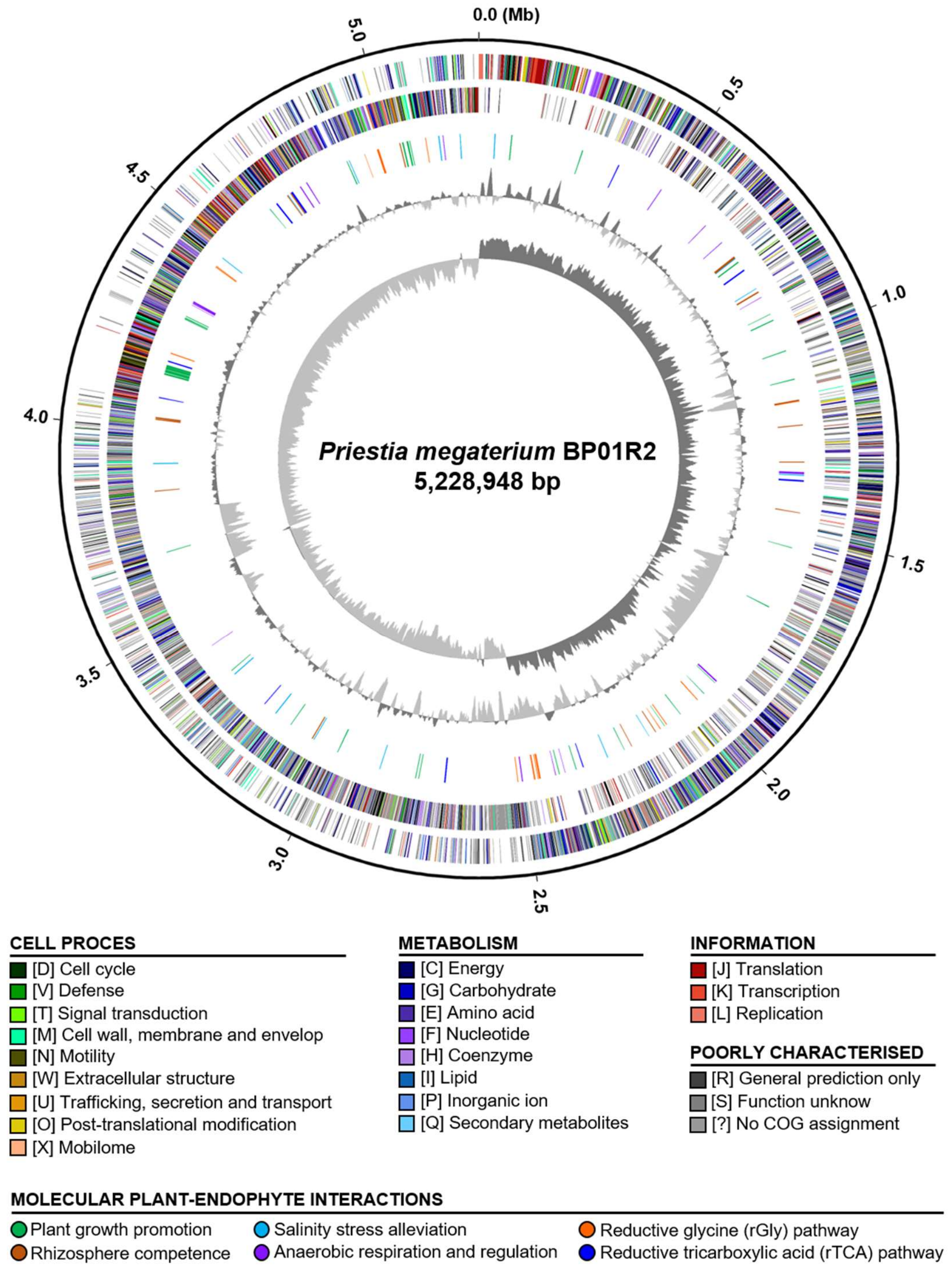

Fig. S19 Genome map of *Priestia megaterium* BP01R2 chromosome. Rings from outside in: (1) Scale marks (Mb). (2 and 3) Forward and reverse strand of coding sequences, respectively. Colour-coded by functional categories (square). (4) Genes associated with identical molecular plant-endophyte interactions. Colour-coded by identified genes involved in metabolism and pathway (circle). See the gene name and locus accession details in Table S6. (5) GC content (dark grey, above average; light grey, average). (6) GC skew (dark grey, positive; light grey, negative).

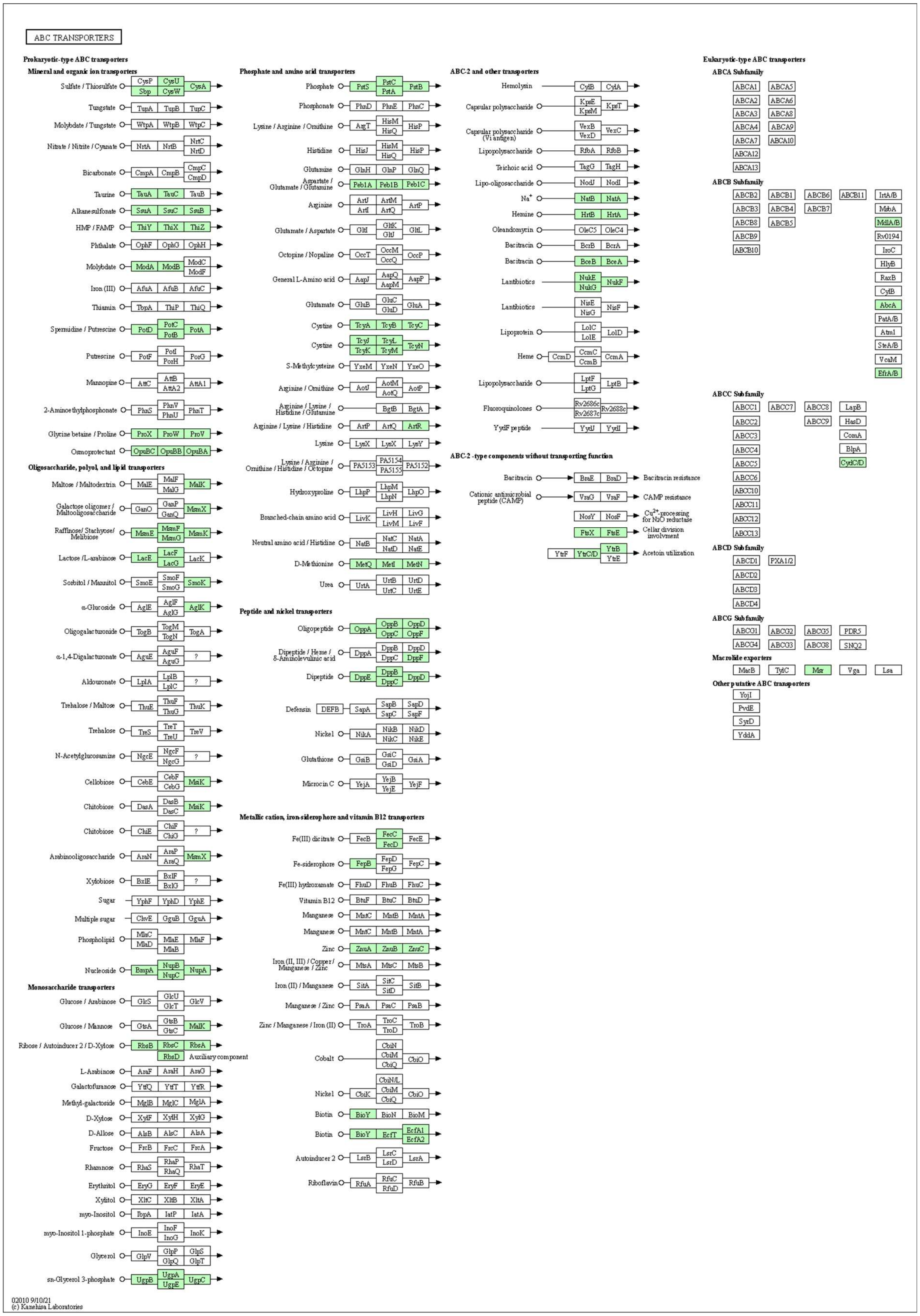

Fig. S20 ABC transporters identified within the genome of BP01R2. The green coloured genes are present in the BP01R2 genome.

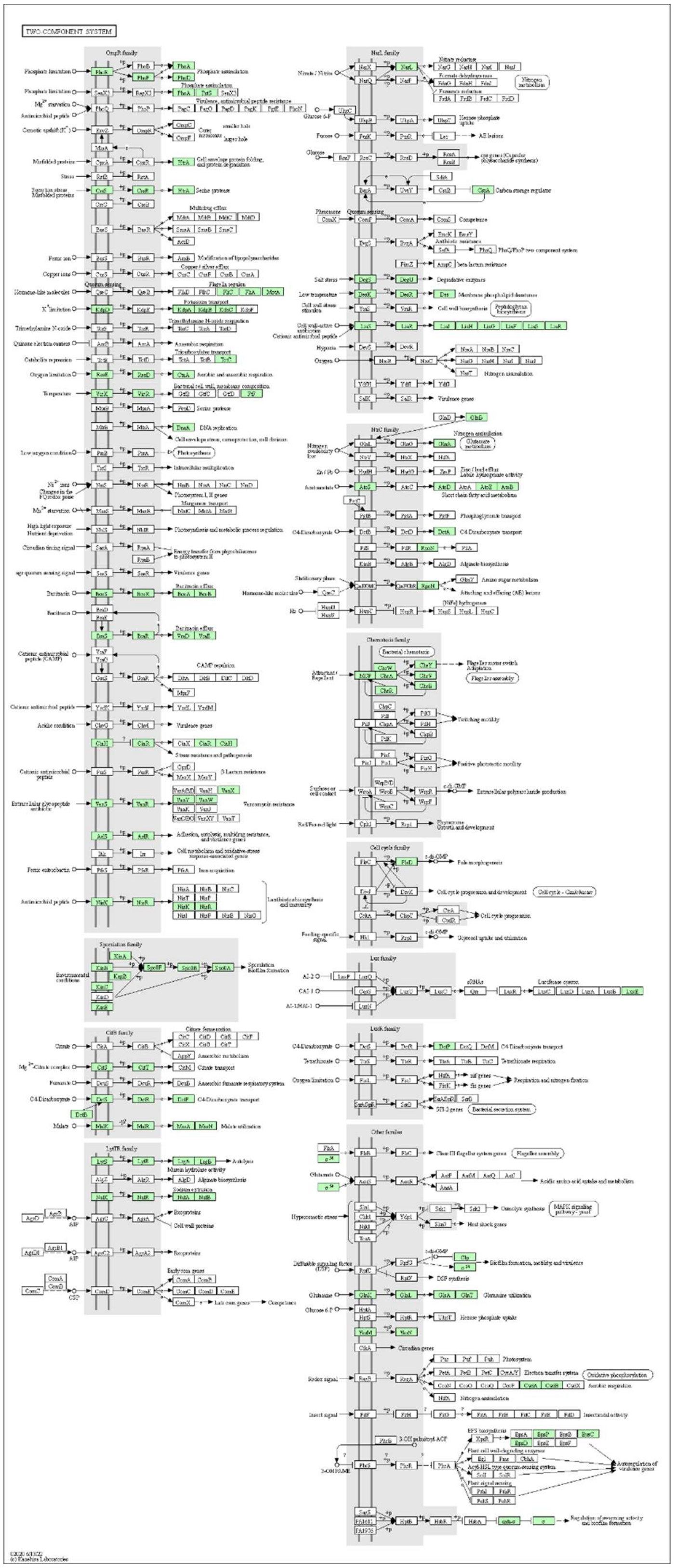

Fig. S21 Two-component systems identified within the genome of BP01R2. The green coloured genes are present in the BP01R2 genome.

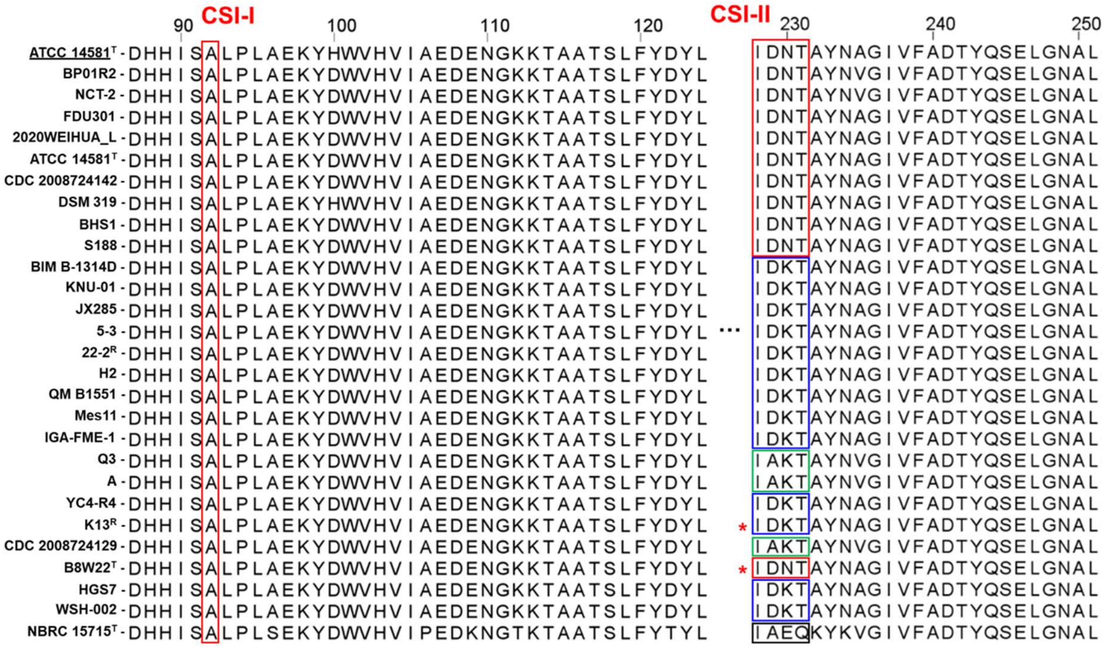

Fig. S22 The multiple sequence alignment analysis of oligoribonuclease NrnB among *Priestia megaterium* and its relatives. CSI-I and CSI-II indicated two conserved signature indels (CSI) previously mentioned by Gupta *et al.* Boxes with different colours tell the amino acid sequence patterns. The *P. megaterium* type strain was underlined. Red asterisks show the type and assigned NCBI representative strains of *P. aryabhattai*. All other strains can be found in Table S1.
